# Ribosome quality control activity potentiates vaccinia virus protein synthesis during infection

**DOI:** 10.1242/jcs.257188

**Published:** 2021-04-28

**Authors:** Elayanambi Sundaramoorthy, Andrew P. Ryan, Amit Fulzele, Marilyn Leonard, Matthew D. Daugherty, Eric J. Bennett

**Affiliations:** 1 Section of Cell and Developmental Biology, University of California, San Diego, La Jolla, CA 92093, USA; 2 Section of Molecular Biology, Division of Biological Sciences, University of California, San Diego, La Jolla, CA 92093, USA

**Keywords:** Integrated stress response, ZNF598, Ribosomes, Ubiquitylation, Vaccinia

## Abstract

Viral infection both activates stress signaling pathways and redistributes ribosomes away from host mRNAs to translate viral mRNAs. The intricacies of this ribosome shuffle from host to viral mRNAs are poorly understood. Here, we uncover a role for the ribosome-associated quality control (RQC) factor ZNF598 during vaccinia virus mRNA translation. ZNF598 acts on collided ribosomes to ubiquitylate 40S subunit proteins uS10 (RPS20) and eS10 (RPS10), initiating RQC-dependent nascent chain degradation and ribosome recycling. We show that vaccinia infection enhances uS10 ubiquitylation, indicating an increased burden on RQC pathways during viral propagation. Consistent with an increased RQC demand, we demonstrate that vaccinia virus replication is impaired in cells that either lack ZNF598 or express a ubiquitylation-deficient version of uS10. Using SILAC-based proteomics and concurrent RNA-seq analysis, we determine that translation, but not transcription of vaccinia virus mRNAs is compromised in cells with deficient RQC activity. Additionally, vaccinia virus infection reduces cellular RQC activity, suggesting that co-option of ZNF598 by vaccinia virus plays a critical role in translational reprogramming that is needed for optimal viral propagation.

## Abstract (precis)

**Summary:** The ribosome-associated quality control factor ZNF598, which senses ribosome collisions, is a host factor necessary for vaccinia viral protein synthesis.

## INTRODUCTION

The task of translating the genetic code into functional proteins is an essential and resource intensive process (Warner, 1999). Dividing cells must double their proteome content prior to cell division to maintain proteome complexity after division. The capacity of ribosomes and the translation-associated machinery can be limiting for this process, as studies in single-cell systems have demonstrated that ribosome content sets cellular proliferation rate (Kafri et al., 2016; Scott et al., 2010; Scott and Hwa, 2011; Scott et al., 2014; Warner, 1999). Interventions that reduce translation speed can constrain cell growth and proliferation (Scott and Hwa, 2011). Given these observations, it is not surprising that one of the most immediate cellular responses to proteotoxic stress is to reduce protein biogenesis output (Costa-Mattioli and Walter, 2020). This reduction in translation has two beneficial outcomes. First, it reduces protein output to limit the production of misfolded, mistranslated or otherwise faulty proteins that require quality control-dependent degradation. Second, limiting translation allows for necessary resource reallocation to mount a successful stress response and restore cellular homeostasis (Costa-Mattioli and Walter, 2020). Viral infection results in proteostasis imbalance as the virus rewires the cellular translation machinery to rapidly generate new viral particles (Walsh and Mohr, 2011).Viral-induced host translation shutoff diverts ∼20–30% of the cellular energy that is normally expended in translating host mRNAs (Buttgereit and Brand, 1995) toward viral protein synthesis. Several viruses, including vaccinia virus, utilize this host shutoff pathway to enhance viral protein synthesis (Abernathy and Glaunsinger, 2015).

Cells combat viral infection through a variety of host defense responses aimed at limiting viral protein production by triggering innate immune signaling pathways (Jan et al., 2016; Stern-Ginossar et al., 2019). One cellular response to proteotoxic stress, including viral infection, is the activation of the integrated stress response (ISR), resulting in eIF2α (also known as EIF2S1) phosphorylation and the subsequent global repression of translation initiation (Costa-Mattioli and Walter, 2020; Liu et al., 2020). Cells utilize the eIF2α kinase, double-stranded RNA (dsRNA)-dependent protein kinase (PKR, also known as EIF2AK2), to inhibit protein biogenesis in the presence of viral dsRNA (McCormack et al., 1992; Williams, 1999). As viral propagation requires the cellular translation machinery, viruses have evolved a myriad set of strategies to combat such host defense mechanisms (Jan et al., 2016). For example, vaccinia virus, a large double-stranded DNA virus that replicates entirely in the cytoplasm of infected cells (Moss, 2013), encodes two different proteins that either bind and occlude double-stranded RNA detection by PKR, or act as a PKR pseudo-substrate (Carroll et al., 1993; Chang et al., 1992; Davies et al., 1992; Meade et al., 2019; White and Jacobs, 2012). The combined impact of these viral proteins is to limit host translation shutdown, highlighting the requirement for sustained ribosome output for rapid viral protein production. These observations also suggest that viral replication requires sustained high levels of active ribosomes to mediate rapid translation of viral mRNAs.

Actively translating ribosomes can become stalled for a sufficient length of time to allow for a trailing ribosome to collide with the stalled ribosome. These ribosome collisions trigger the ribosome-associated quality control (RQC) pathway that acts to ubiquitylate and destroy the attached nascent chain as well as catalyze ribosome subunit splitting and mRNA disengagement (Inada, 2020; Joazeiro, 2019). These outcomes limit the abundance of potentially toxic truncated translation products and recycle ribosomes to re-enter the translation cycle. The inability to recognize collided ribosomes and initiate the RQC pathway results in ribosomal readthrough of stall-inducing sequences and codon frameshifting (Joazeiro, 2019; Juszkiewicz and Hegde, 2017; Juszkiewicz et al., 2020a; Sundaramoorthy et al., 2017; Wang et al., 2018). Additionally, a failure to recycle collided ribosomes can deplete the active ribosome pool and result in reduced tissue function (Liakath-Ali et al., 2018; Mills and Green, 2017; Mills et al., 2016). Rapid viral propagation requires efficient translation of viral mRNAs, a task made more difficult because viruses often feature complex secondary structures in their mRNA transcripts (Smyth et al., 2018) and disrupt tRNA pools due to mass production of comparatively small proteomes (Nunes et al., 2020). These observations suggest that virally infected cells may be especially dependent on robust RQC function to maintain sufficient levels of available ribosomes necessary for viral proliferation.

In mammals, the RQC pathway requires site-specific regulatory ribosomal ubiquitylation (RRub), catalyzed by the ubiquitin ligase ZNF598 (Juszkiewicz and Hegde, 2017; Matsuo et al., 2017; Sundaramoorthy et al., 2017). ZNF598 ubiquitylates the 40S ribosomal proteins eS10 and uS10 (RPS10 and RPS20, respectively, using older nomenclature) at precise lysine residues, and either loss of ZNF598 or mutations that block eS10 or uS10 ubiquitylation result in RQC failure and ribosomal readthrough of stall-inducing sequences (Garshott et al., 2020; Juszkiewicz and Hegde, 2017; Sundaramoorthy et al., 2017). Thorough biochemical and genetic studies in multiple organisms have delineated a growing list of molecular constituents within the RQC pathway that act on collided ribosomes (Inada, 2020; Joazeiro, 2019). Recent studies have uncovered cellular signaling pathways that are induced by collisions that both mount a transcriptional response and result in translation initiation inhibition by multiple routes (Hickey et al., 2020; Juszkiewicz et al., 2020a; Meydan and Guydosh, 2021a; Sinha et al., 2020; Tollenaere et al., 2019; Vind et al., 2020a; Wu et al., 2020). How viruses utilize various RQC components for viral propagation or are impacted by RQC dysfunction remains largely unexplored.

Here, we utilize vaccinia virus to interrogate the interplay between the RQC pathway and viral protein synthesis. Vaccinia virus is known to manipulate host translation shutdown and has a large proteome, allowing for more detailed analysis of how fluctuations in translation capacity affect viral replication (Dhungel et al., 2020). Indeed, previous results have revealed that ZNF598 and uS10 ubiquitylation is needed for optimal vaccinia virus replication (DiGiuseppe et al., 2018). Here, we show that chronic loss of ZNF598 function, either through ZNF598 knockout (KO) or specific mutations within uS10 that prevent ubiquitylation, results in widespread reduction of nearly all viral protein production with little effect on viral transcription. Furthermore, vaccinia virus infection reduces overall cellular RQC activity, indicating that viral protein production depletes a critical RQC factor. Taken together, our findings lead us to conclude that vaccinia virus infection induces ribosome collisions and that the RQC pathway, through ZNF598 and its substrate uS10, rescues stalled ribosomes from non-functional translation events and recycles ribosomal subunits for optimal translation of viral mRNAs.

## RESULTS

### Ribosome collisions occur during integrated stress response activation in the absence of eIF2α phosphorylation

**Fig. 1.**
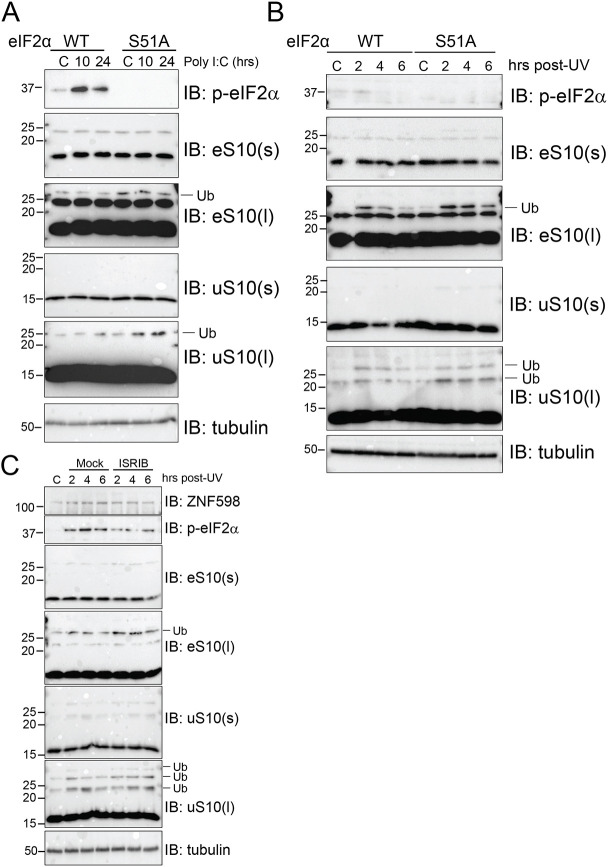
**Loss of eIF2α phosphorylation leads to higher frequency ribosome collisions during proteotoxic stress.** (A) MEFs derived from wild-type (WT) or S51A mutant eIF2α animals were mock transfected (C) or transfected with poly(I:C), and cells were harvested at the indicated time points post transfection. Whole-cell extracts were analyzed using SDS–PAGE and were immunoblotted (IB) with antibodies to detect the indicated proteins (p-eIF2α, phosphorylated eIF2α; l, long exposure; s, short exposure; Ub, ubiquitylated protein). (B) MEFs of the indicated genotypes were unexposed (C) or exposed to 200 J/m^2^ UV and allowed to recover for the indicated times prior to harvesting. Cells were harvested at the indicated time points, whole-cell extracts were run on SDS–PAGE gels and immunoblotted with antibodies against the indicated proteins. (C) 293T cells were exposed to 200 J/m^2^ UV and allowed to recover in normal medium or in the presence of 200 nM ISRIB. Cells were harvested at the indicated time points and total lysates were resolved using SDS–PAGE and immunoblotted using antibodies against the indicated proteins. Size markers indicate molecular masses in kDa. Blots shown are representative of *n*=2 experiments.

Viral replication consumes the host translation machinery, and host defense strategies often target the translation machinery to combat viral propagation (Jan et al., 2016). The observation that viruses often employ countermeasures to maintain high rates of translation suggests that any loss in available ribosomes may be particularly deleterious to viral replication. RQC pathway activation upon ribosome collisions acts to recycle stalled ribosomal subunits for reuse during translation (Joazeiro, 2019). As such, viruses may depend on the RQC machinery to rapidly liberate collided ribosomes during viral mRNA translation. Many viruses, including vaccinia virus, antagonize PKR-dependent eIF2α phosphorylation to prevent host shutdown of translation initiation (Dhungel et al., 2020; Meade et al., 2019). To examine if ribosome collisions occur at a higher frequency during innate immune or ISR activation when eIF2α phosphorylation is compromised, we utilized mouse embryonic fibroblasts (MEFs) containing either wild-type or S51A mutant eIF2α that cannot be phosphorylated (Scheuner et al., 2001). We used polyinosinic:polycytidylic acid [poly(I:C)] transfection to mimic viral infection-mediated activation of innate immune signaling pathways. Poly(I:C) transfection resulted in robust eIF2α phosphorylation in wild-type MEFs and failed to induce eS10 or uS10 ubiquitylation, which are markers for ribosome collisions (Fig. 1A). In contrast, poly(I:C) transfection in cells unable to repress translation through eIF2α phosphorylation resulted in a time-dependent increase in uS10 ubiquitylation, indicating elevated levels of ribosome collisions (Fig. 1A). To further examine the impact of compromised eIF2α phosphorylation on ribosome collisions, we exposed cells to UV, which induces ribosome collisions and the ISR. As expected, UV exposure resulted in ZNF598-dependent eS10 and uS10 ubiquitylation despite enhanced eIF2α phosphorylation (Garshott et al., 2020). We observed that uS10 ubiquitylation was enhanced in eIF2α-mutant MEFs upon UV exposure compared to that in wild-type MEFs, indicating that an inability to repress translation initiation upon proteotoxic stress increases ribosome collision frequency (Fig. 1B). Consistent with the notion that antagonizing eIF2α signaling results in enhanced ribosome collisions, addition of ISRIB, which blocks the inhibitory activity of phospho-eIF2α (Sidrauski et al., 2015), enhanced the UV-dependent eS10 and uS10 ubiquitylation (Fig. 1C). This observation indicates that an inability to downregulate translation via eIF2α phosphorylation results in an elevated frequency of ribosome collisions upon UV exposure. Taken together, these results suggest that antagonizing eIF2α phosphorylation during proteotoxic stress enhances ribosome collisions.

### Vaccinia virus replication is inhibited upon loss of ZNF598 or uS10 ubiquitylation

**Fig. 2.**
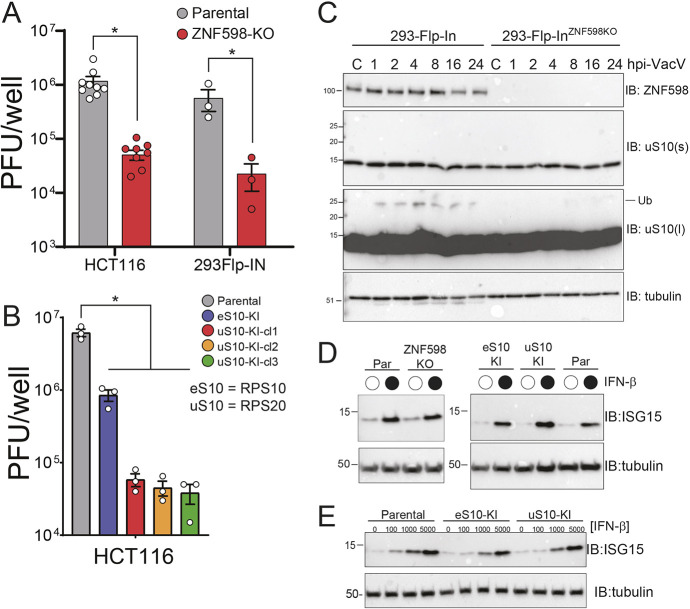
**Vaccinia virus replication is attenuated in cells unable to mount an RQC response.** (A) ZNF598 KO or parental HCT116 or 293Flp-IN cells were infected with vaccinia virus (10,000 PFU/well, MOI ∼0.04). Cells were collected 24 h post infection, and viral titers were determined using a plaque assay. Data are presented as the mean±s.e.m. of three experiments, with individual points shown. *P<0.05 (one-way ANOVA with adjustment for multiple comparisons using Tukey's method). (B) HCT116 cells containing mutant ribosomal proteins eS10 (K138R, K139R; eS10-KI) or uS10 (K4R, K8R; uS10-KI), or parental HCT116 cells were infected with vaccinia virus (10,000 PFU/well, MOI ∼0.04). Cells were collected 24 h post infection, and viral titers were determined using a plaque assay. Three individual clones (cl1, cl2 and cl3) of uS10-KI cells were tested. Data are presented as the mean±s.e.m. of three experiments, with individual points shown. *P<0.05 (one-way ANOVA with adjustment for multiple comparisons using Tukey's method). (C) Parental or ZNF598 KO 293Flp-IN cells were mock infected (C) or infected with vaccinia virus (VacV; MOI of 5), and cells were collected at the indicated time points (hpi, hours post infection) then analyzed using SDS–PAGE and immunoblotted (IB) with antibodies against the indicated proteins (l, long exposure; s, short exposure; Ub, ubiquitylated protein). (D,E) The indicated cell lines (Par, parental) were either untreated (open circle) or treated (filled circle) with 1000 U of IFN-β for 16 h (D) or treated with the indicated concentration of IFN-β (100 U, 1000 U and 5000 U) for 16 h (E), and cells were harvested and analyzed by SDS–PAGE and immunoblotting. In C–E, size markers indicate molecular masses in kDa. Blots shown are representative of *n*=2 experiments.

We reasoned that viral-mediated proteotoxic stress, combined with viral antagonism of eIF2α phosphorylation, might render RQC activity important for viral replication. To directly test if vaccinia virus replication is impacted by loss of RQC activity, parental and ZNF598 KO cells were infected with vaccinia virus and viral titers were determined 24 h post infection. Vaccinia virus replication was repressed more than 10-fold in two independent ZNF598 KO cell lines, which is consistent with previous results (DiGiuseppe et al., 2018) (Fig. 2A). We previously generated and characterized point-mutant knock-in cell lines in which critical lysine residues (K138 and K139 within eS10, and K4 and K8 within uS10) in eS10 or uS10 that are ubiquitylated by ZNF598 and are required for RQC activity are mutated to arginine (Garshott et al., 2020). Vaccinia virus infections in mutant eS10 or uS10 cell lines resulted in 10–100-fold less viral replication compared to infections in parental cells (Fig. 2B). Interestingly, multiple clones of uS10 point-mutant lines displayed a more profound defect in vaccinia virus replication compared to the eS10 mutant cell line across several experiments. These results confirm that vaccinia virus requires ZNF598-mediated ribosomal ubiquitylation for optimal viral replication (DiGiuseppe et al., 2018). Consistent with the viral titer data, vaccinia virus infection resulted in enhanced uS10 ubiquitylation throughout the infection timecourse, indicating that vaccinia virus infection induces ribosome collisions during cellular replication (Fig. 2C; Fig. S1A,B). Although eS10 ubiquitylation was also stimulated at early time points, it was less consistently ubiquitylated compared to uS10 upon vaccinia virus infection (Fig. S1B). Previous studies have demonstrated that ZNF598 utilizes an internal polyproline motif to engage in translational repression of AU-rich cytokine transcripts by binding to the GIGYF–4EHP complex (Tollenaere et al., 2019). ZNF598 also represses expression of interferon stimulated genes (ISGs), which are critical for pathogen defense responses (DiGiuseppe et al., 2018). Transient loss of ZNF598 has been shown to be broadly antiviral due to the de-repression of anti-inflammatory cytokines (DiGiuseppe et al., 2018; Tollenaere et al., 2019). Furthermore, loss of ZNF598 expression enhances RIG-I (DDX58)-mediated signaling and the protein abundance of several ISGs (Wang et al., 2019). However, all these ZNF598-dependent effects on ISG expression do not depend on its ligase activity, and it is unclear if limiting RQC activity itself alters ISG expression. Based on these previous studies, we monitored ISG stimulation upon interferon-β (IFN-β) addition to examine if interferon signaling was constitutively activated in our RQC mutant cell lines. The basal levels of ISG15 were not elevated in ZNF598 KO cells or in our ribosome point-mutation knock-in cells compared to levels in parental cells (Fig. 2D,E). Moreover, ISG expression in these RQC-deficient cells lines mirrored that in wild-type cells, with IFN-β addition resulting in robust ISG15 production in all cell lines (Fig. 2D,E). These results, combined with previous results (DiGiuseppe et al., 2018) and the observation that vaccinia virus replication is mostly insensitive to interferon addition (Smith et al., 2018), argue that the loss of RQC pathway function is responsible for the observed vaccinia virus replication defect in ZNF598 KO cell lines.

### ZNF598-mediated uS10 ubiquitylation is needed for optimal vaccinia virus protein synthesis

**Fig. 3.**
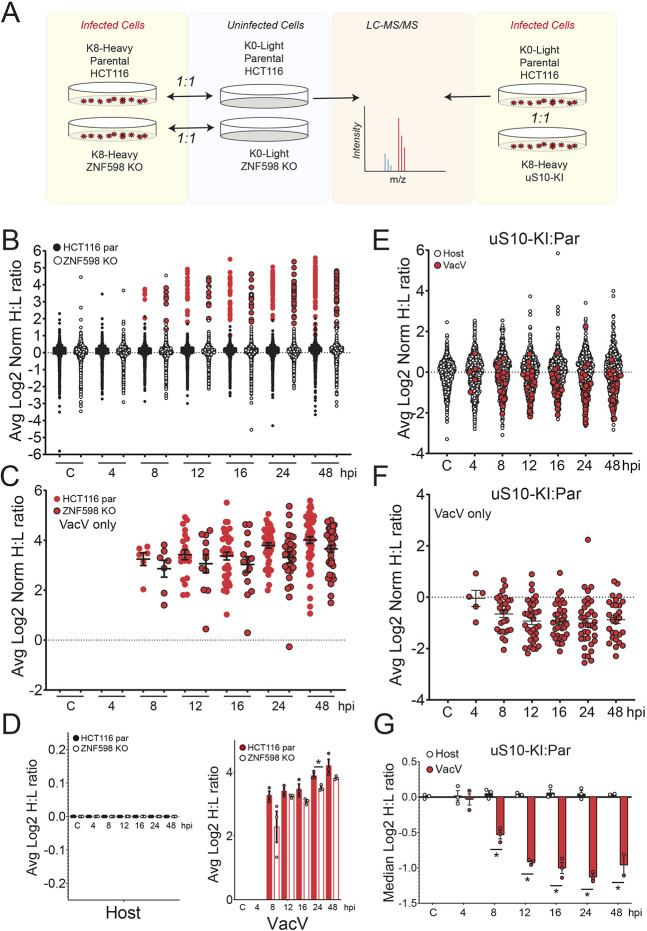
**Proteomic characterization of vaccinia virus protein production in RQC-deficient cells.** (A) Schematic showing the labeling strategy for the SILAC experiments. (B) Heavy-labeled parental (par; filled circles) or ZNF598 KO (open circles) HCT116 cells were infected with vaccinia virus (MOI=5), collected at the indicated time points post infection (hpi, hours post infection), and mixed 1:1 with light-labeled uninfected cells of the same genotype. Samples were analyzed by mass spectrometry, and normalized log_2_ heavy to light (H:L) ratios were determined for host (black) and vaccinia virus (VacV; red) proteins (Table S1). The mean normalized log_2_ H:L ratio for proteins across three replicate experiments is depicted. (C) Normalized log_2_ H:L ratios of VacV proteins from parental (red circles, no border) and ZNF598 KO (red circles, black border) cells infected with VacV (MOI=5) at the indicated time points (Table S2). The mean normalized log_2_ H:L ratio for proteins across three replicate experiments is depicted. Black bars denote the mean of all VacV H:L ratios at a given time point with error bars representing s.e.m. (D) The mean normalized log_2_ H:L ratio for host (left) or VacV (right) proteins in parental (filled black or red bars) or ZNF598 KO (unfilled bars) cells at each time point. Error bars denote s.e.m. for triplicate experiments. *P<0.05 (two-tailed, unpaired Student's *t*-test). (E,F) Light-labeled parental cells and heavy-labeled uS10-KI cells were infected with vaccinia virus, collected at the indicated time points post infection, and mixed 1:1. Samples were analyzed by mass spectrometry, and mean normalized log_2_ H:L ratios were determined for (E) host (open) and VacV (filled red) proteins (Table S3) or (F) just VacV proteins (Table S4). The mean normalized log_2_ H:L ratio for proteins across three replicate experiments is depicted. Black bars in F denote the mean of all VacV H:L ratios at a given time point with error bars representing s.e.m. (G) The median normalized log_2_ H:L (parental:uS10-KI) ratio for host (black bars) or VacV (red bars) proteins at each time point. Error bars denote s.e.m. for triplicate experiments. *P<0.05 (two-tailed, unpaired Student's *t*-test).

Based on our data and previous reports, it is possible that translation of a specific subset of essential vaccinia virus mRNAs are specifically inhibited upon loss of ZNF598-mediated uS10 ubiquitylation, as has been proposed previously (DiGiuseppe et al., 2018). Alternatively, defective RQC pathway activity could broadly impact ribosome availability, which would result in reduced translation of many viral mRNAs. To differentiate between these possibilities, we performed stable isotope labeling of amino acids in culture (SILAC)-based quantitative proteomics experiments upon infection with vaccinia virus in parental and ZNF598 KO cells. Heavy (Lys8)-labeled parental or ZNF598 KO cells were infected with vaccinia virus at a multiplicity of infection (MOI) of five, and cells were harvested at seven time points post infection then mixed with light (Lys0)-labeled uninfected cells of the same genotype (Fig. 3A). We quantified the abundance of 63 viral proteins in at least a single time point (Tables S1,S2). As expected, viral protein abundance increased over time, with the most viral proteins detected 48 h after infection (Fig. 3B,C). An observable reduction in the mean vaccinia virus protein abundance in ZNF598 KO cells compared to parental controls could be detected 8 h post infection (Fig. 3C,D). No difference in overall host protein abundance was observed between ZNF598 KO and parental cells (Fig. 3D; Table S1). These results are consistent with our viral titer assays in that viral protein production was repressed in ZNF598 KO cells. To verify that this result was due to an inability to mount an RQC response, we performed a similar proteomics experiment in parental and uS10 point-mutant knock-in cells (uS10-KI). Because only a single time point showed a statistically significant difference in the ZNF598 KO cells due to variance observed by comparing ratios of ratios, we modified the labeling scheme for this series of experiments such that heavy-labeled uS10-KI cells and light-labeled parental cells were both infected at an MOI of 5 and the two cellular genotypes were collected over a timecourse post infection and mixed (Fig. 3A). This mixing scheme allows direct comparison of differences between genotypes in the same sample. As observed in ZNF598 KO cells, overall host protein abundance was unchanged upon infection (Table S3), but vaccinia virus protein production was broadly repressed in uS10-KI cells beginning 8 h post infection (Fig. 3E–G; Tables S3,S4). Consistent with a previous report, a small number of host proteins displayed vaccinia virus- and genotype-specific abundance changes (Soday et al., 2019). However, despite these changes, overall host protein abundance was not impacted by vaccinia virus infection (Fig. 3G; Table S3). Combined, our data suggests that an inability to ubiquitylate collided ribosomes results in a functional ribosomal imbalance, leading to compromised viral protein production.

### Translation of mRNAs containing a 5**′** UTR poly(A) sequence is not specifically impacted by loss of ZNF598 activity

**Fig. 4.**
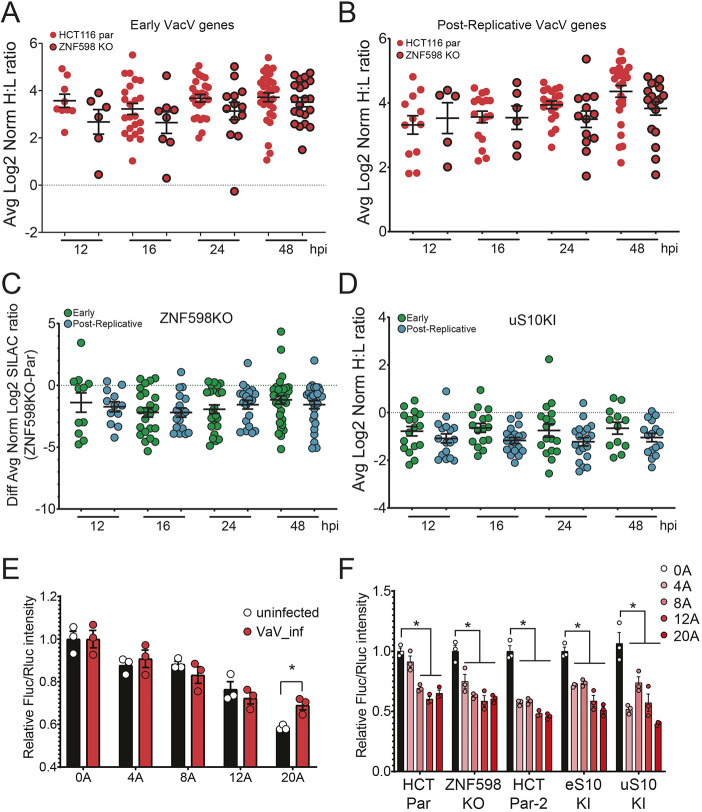
**RQC deficiency does not specifically impact translation of mRNAs containing a 5′ UTR poly(A) sequence.** (A,B) Vaccinia virus (VacV) proteins were binned as (A) early or (B) post-replicative, and the normalized log_2_ heavy to light (H:L) ratios of VacV proteins from parental (par; red circles, no border) or ZNF598 KO (red circles, black border) HCT116 cells infected with VacV (MOI=5) are compared at the indicated time points (hpi, hours post infection). The mean normalized log_2_ H:L ratio for proteins across three replicate experiments is depicted. Black bars denote the mean of all VacV H:L ratios at a given time point with error bars representing s.e.m. (C) The difference in mean normalized log_2_ H:L ratios for VacV early proteins (green circles) or post-replicative proteins (blue circles) between infected ZNF598 KO and parental cells is depicted. Black bars denote the mean of all H:L ratios at a given time point with error bars representing s.e.m. All mean comparisons are not significant (one-way ANOVA). (D) The mean normalized log_2_ H:L (uS10-KI:parental) ratios of VacV early proteins (green circles) or post-replicative proteins (blue circles) is depicted. Black bars denote the mean of all H:L ratios at a given time point with error bars representing s.e.m. All mean comparisons are not significant (one-way ANOVA). (E) Relative luminescence intensities of firefly luciferase (Fluc) reporters containing the indicated length of poly(A) sequence (0A–20A) immediately before the start codon for firefly luciferase, compared to Renilla luciferase (Rluc) controls without poly(A) sequences, is shown for uninfected (black bars) and VacV infected (red bars) cells. Mean±s.e.m. for triplicate experiments. *P<0.05 (two-tailed, unpaired Student's *t*-test). (F) Relative luminescence intensities of firefly luciferase reporters containing the indicated length of poly(A) sequence, as compared to Renilla luciferase controls, is shown for transfections into the indicated cell types (HCT Par, parental line for the ZNF598 KO; HCT Par-2, parental line for the eS10-KI and uS10-KI lines). Mean±s.e.m. for triplicate experiments. *P<0.05 (two-tailed, unpaired Student's *t*-test).

Consistent with previous reports, we found that vaccinia virus replication was reduced in cells lacking the ability to ubiquitylate ribosomes during RQC events (DiGiuseppe et al., 2018). One possible proposed mechanism to explain the vaccinia virus dependence on RQC is that translation of post-replicative vaccinia virus mRNAs containing non-templated poly(A) sequences of various length within their 5′ UTR is specifically impacted by loss of ZNF598 activity. Contrary to this model, we found that viral protein production was broadly repressed in ZNF598 KO or uS10-KI cells. To more directly investigate if RQC deficiency specifically impacted vaccinia virus protein production from mRNAs containing poly(A) sequences in their 5′ UTRs, we separated our vaccinia virus proteomic data into proteins that are expressed early, before viral DNA replication, and those that are classified as intermediate or late expressed proteins (collectively referred to as post-replicative), based on previous transcriptional profiling studies (Yang et al., 2010, 2011). Both early and post-replicative vaccinia virus proteins were repressed in ZNF598 KO and uS10-KI cells (Fig. 4A–D). Direct comparison of early versus post-replicative viral protein abundance did not reveal any significant difference at any time point post infection in either ZNF598 KO or uS10-KI cells (Fig. 4C,D). This result suggests that viral protein production is repressed irrespective of the presence of 5′ UTR poly(A) sequences in RQC-deficient cells. To probe this hypothesis further, we constructed reporter plasmids containing poly(A) sequences of varying lengths immediately prior to the start codon of the firefly luciferase coding sequence. Consistent with previous reports, firefly luciferase protein production decreased as the 5′ UTR poly(A) sequence length increased, as compared to a control Renilla luciferase reporter (Fig. 4E) (Dhungel et al., 2017). Performing a similar experiment in vaccinia virus-infected cells revealed the same poly(A) length-dependent decrease in firefly luciferase protein production. However, vaccinia virus infection resulted in enhanced firefly luciferase protein levels with the longest poly(A) sequence compared to levels in uninfected cells (Fig. 4E). This is consistent with previous reports showing that virus-mediated phosphorylation of the 40S ribosomal protein RACK1 facilitates translation of late viral mRNAs with 5′ UTR poly(A) sequences (Jha et al., 2017). Transfection of these reporters into ZNF598 KO, uS10-KI or eS10-KI cells in the absence of viral infection resulted in a similar poly(A)-dependent repression of firefly luciferase protein levels, indicating that a loss of RQC activity does not allow for enhanced protein production for mRNAs containing a 5′ UTR poly(A) sequence (Fig. 4F). Previous studies have demonstrated that loss of ZNF598 results in enhanced protein production downstream of poly(A) sequences within the coding sequence of reporter mRNAs (Juszkiewicz and Hegde, 2017; Sundaramoorthy et al., 2017). This is due to an inability to sense collided ribosomes during translation elongation in RQC-deficient cell lines. While recent reports have demonstrated that elongation collisions can result in feedback inhibition of translation initiation by various mechanisms (Hickey et al., 2020; Juszkiewicz et al., 2020a; Meydan and Guydosh, 2020; Sinha et al., 2020; Vind et al., 2020b; Wu et al., 2020; Yan and Zaher, 2021), these pathways act largely in a manner that does not require ZNF598 activity nor ribosome ubiquitylation. Consistent with the lack of an established direct role for ZNF598 or uS10 or eS10 ubiquitylation during ribosome scanning prior to elongation, our data indicates that mRNAs containing non-coding poly(A) sequences within the 5′ UTR, while broadly repressive, are not translated more efficiently in cells with defective RQC activity.

### Dual RNA-seq of vaccinia infection reveals host and vaccinia transcriptional responses to be independent of RQC

**Fig. 5.**
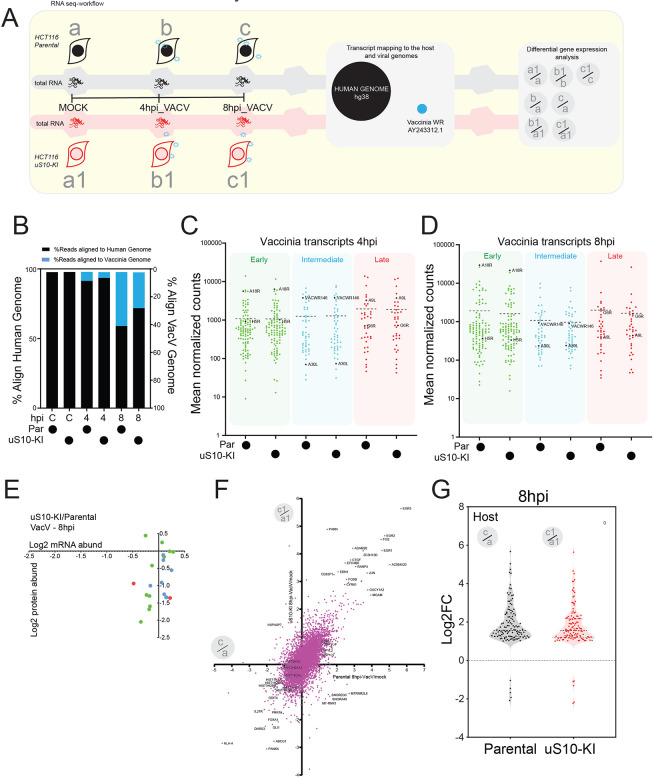
**RNA-seq identifies intact viral and host transcriptional response in uS10-KI cells.** (A) Schematic depicting the RNA-seq experimental design and analysis. Individual RNA library preparation time points are shown as a, b and c for parental HCT116 cells, and as a1, b1 and c1 for uS10-KI HCT116 cells (hpi, hours post infection; VacV, vaccinia virus). (B) Percentage mRNA alignment to the human (black bars) or VacV (blue bars) genome in parental (Par) or uS10-KI cells (C, mock-infected control sample). (C,D) Reads aligned to the VacV genome were analyzed using DESeq2, and the mean normalized counts of VacV genes, classified as early (green), intermediate (blue) or late transcribing (red), are depicted in the indicated cell lines at (C) 4 hpi. (Table S7) or (D) 8 hpi (Table S8). Dashed lines indicate the mean of the normalized counts of the plotted genes. Named genes show a similar trend of gene expression across the genotypes, across temporal expression patterns, and are plotted in a heatmap in Fig. S2B. (E) Plot of the log_2_-transformed ratio (uS10-KI:parental) of protein abundance (as assayed using SILAC) versus mRNA abundance (RNA-seq mean read counts) for VacV genes at 8 hpi (Table S9). Circle color indicates mRNA expression classification (green, early; blue, intermediate; red, late). (F) Scatter plot of log_2_ fold change (8 hpi/mock) of mRNA expression for host genes in parental cells (*x* axis) versus uS10-KI cells (*y* axis) (*n*=13,759) (Tables S10, S11). Selected genes are labeled. (G) Violin plot showing the frequency distribution of log_2_ fold-change (FC) ratios (8 hpi:mock) of the 122 common differentially expressed host genes in parental and uS10-KI cell lines when comparing uninfected cells with those at 8 h post infection.

It is possible that our proteomics results demonstrating broad repression of viral protein production in RQC-deficient cell lines were due to defects in viral mRNA transcription. To directly address this possibility, we performed RNA-seq analysis of mock-treated and vaccinia virus-infected parental or uS10-KI cells at 4 and 8 h post infection (Fig. 5A). The sequencing experiment generated ∼15 million reads per sample per time point, totaling 270 million reads (Fig. S2A). Expression of ∼12,000 human genes was quantitatively documented across each time point, and 200 vaccinia genes were sampled in infected samples (Tables S5–S8). The reads were aligned in parallel to human and vaccina genomes to investigate the transcriptional dynamics during infection. More than 97% of reads from mock-treated samples aligned to the human genome, whereas in samples from vaccinia virus-infected cells at 4 h post infection, 91–93% of reads aligned to the host genome (Fig. 5B). In parental cells, the percentage of reads mapped to the host genome further decreased to 59% at 8 h post infection, demonstrating the expected increase in viral mRNA abundance during infection (Fig. 5B). The percentage of reads aligned to the vaccinia genome increased from 6% at 4 h to 39% 8 h post infection in parental cells and from 4% to 26% in uS10-KI cells (Fig. 5B). Previous reports using HeLa cells infected with vaccinia virus have identified 70% of mRNA at 8 h post infection as being of viral origin (Yang et al., 2015).

We analyzed the transcriptional dynamics of vaccinia genes at 4 and 8 h post infection within parental and uS10-KI cells. We observed no difference in vaccinia virus mRNA levels 4 h post infection between parental and uS10-KI cells (Fig. 5C; Table S7). There was a small but noticeable overall decrease in the mean vaccinia virus mRNA abundance in uS10-KI cells compared to that in parental cells 8 h after vaccinia virus infection (Fig. 5D; Table S8). This decrease is consistent with our data showing a defect in vaccinia virus viral propagation in uS10-KI cells. Analysis of genes differentially expressed between parental and uS10-KI cells revealed 39 vaccinia virus mRNAs (out of 200) with reduced expression in uS10-KI cells at 8 h post infection (Table S8). Comparison of early versus post-replicative vaccinia virus mRNA abundance revealed no differences between parental and uS10-KI cells at either time point (Fig. 5C,D; Tables S7,S8). Overall, our analysis indicates that, although a small number of vaccinia virus genes show reduced mRNA levels at 8 h post infection, there is no evidence of widespread alternations in vaccinia virus gene transcription in uS10-KI cells (Fig. S2B; Tables S5–S8). A direct comparison of protein and mRNA abundance for vaccinia virus genes with matched proteomics and RNA-seq data 8 h post infection in parental and uS10-KI cells revealed protein abundance to be reduced more than mRNA abundance for these vaccinia virus genes irrespective of their temporal mRNA expression classification (Fig. 5E; Table S9). These results are consistent with a model in which the observed difference in viral protein production in our proteomics experiments is due to a posttranscriptional mechanism.

We next analyzed the host transcriptional response to vaccinia infection and compared gene expression changes as a function of viral infection within the same cell type. Clear host transcriptional responses could be observed 8 h after infection, including the documented MAPK-based activation of FOS, EGR1, and c-JUN gene expression, in both parental and uS10-KI cells (Fig. 5F, Tables S10, S11). Furthermore, the overall transcriptional response to vaccinia virus infection was highly similar when comparing parental and uS10-KI cells (Fig. 5F,G; Fig. S2C–E; Tables S12,S13). Examining genes characterized by both a statistically significant change in abundance and whose abundance increased or decreased by more than twofold, we observed 332 (285 genes with increased expression, 47 genes with decreased expression) and 228 (203 genes with increased expression, 25 genes with decreased expression) host genes with differential expression compared to uninfected controls in parental and uS10-KI cells, respectively (Tables S10,S11). The set of common upregulated genes was enriched in genes involved in known viral-induced and pro-inflammatory signaling pathways. Indeed, known virus-induced transcription factors like RELB and EGR1 were similarly induced by vaccinia virus infection in both parental and uS10-KI cells (Fig. S2F).

Although the overwhelming majority of transcripts were altered similarly between parental and uS10-KI cells in response to vaccinia virus infection, our analysis revealed 137 and 93 genes that were differentially expressed between parental and uS10-KI cells at 4 and 8 h post infection, respectively (Tables S14,S15). Pathway analysis of these genes did not reveal clear enrichment of known pathways or specific gene ontology categories. We also analysed differential gene expression in mock-treated cells, comparing between the two cell lines, and identified 112 genes (Table S16). These baseline gene expression differences are unlikely to account for the observed vaccinia virus proliferation defect in uS10-KI cells as only one of these genes was found to be differentially expressed upon vaccinia virus infection in parental cells or uS10 cells. Combined, our analyses revealed that the RQC-deficient uS10-KI cells display similar host and viral transcript levels upon vaccinia virus infection. Given the wide fluctuation in viral protein levels in these cells, we propose that a posttranscriptional mechanism governs the loss of viral protein expression.

### Vaccinia virus infection limits cellular RQC activity

**Fig. 6.**
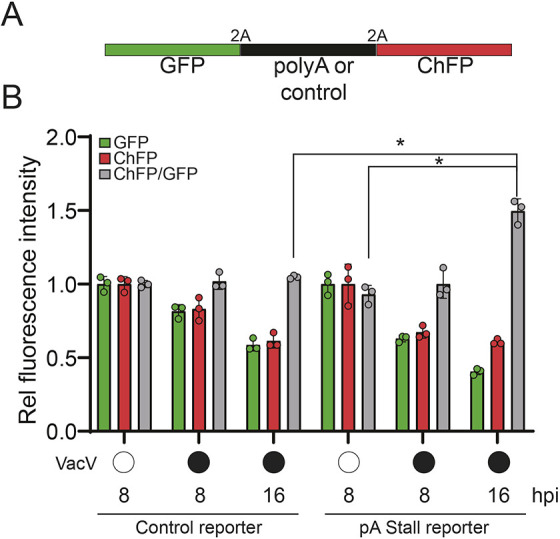
**Vaccinia virus infection alters cellular RQC activity.** (A) Schematic of the fluorescent stall reporter plasmid with either a control or poly(A) sequence in the middle coding region. 2A denotes ribosomal skipping sequence. (B) The relative GFP and ChFP fluorescence intensities, and the ChFP/GFP intensity ratio in cells transfected with either the control or poly(A)-containing stall reporter plasmids are depicted for uninfected (open circles) or vaccinia virus (VacV)-infected (filled circles) cells at the indicated time points post infection (hpi, hours post infection). Mean±s.d. of triplicate experiments. *P<0.05 (two-tailed, unpaired Student's *t*-test).

Our data suggest that vaccinia virus infection enhances the frequency of ribosome collisions and that an inability to clear these collisions compromises vaccinia virus mRNA translation. These results support a model in which vaccinia virus replication relies on a cellular pool of available ribosomes that becomes depleted in RQC-defective cells. To probe this possible model, we utilized a well-characterized fluorescent RQC reporter in which the genes encoding GFP and ChFP are separated by a third coding sequence containing a decoded poly(A) sequence (Fig. 6A) (Juszkiewicz and Hegde, 2017; Sundaramoorthy et al., 2017). Cells with reduced ZNF598 protein expression or containing eS10 or uS10 mutations in ubiquitin-acceptor lysine residues display enhanced ChFP expression downstream of the poly(A) sequence, relative to upstream GFP expression, because ribosomes are unable to be cleared from poly(A)-induced stalls and eventually decode the downstream ChFP (Garshott et al., 2020; Juszkiewicz and Hegde, 2017; Sundaramoorthy et al., 2017). Vaccinia virus infection of cells expressing a control reporter lacking a poly(A) sequence resulted in a time-dependent decrease in both GFP and ChFP expression indicative of host-dependent translation repression upon vaccinia virus infection (Fig. 6B). Infection of cells expressing the poly(A)-containing stall reporter also resulted in translational repression of GFP expression. However, vaccinia virus infection also resulted in elevated ChFP expression relative to GFP at 16 h post infection (Fig. 6B). This result suggests that cellular RQC activity is decreased upon vaccinia virus infection and that vaccinia virus viral factories may be titrating ZNF598 activity away from the cellular pool. Alternatively, viral infection could simply overwhelm the limiting RQC capacity in cells due to enhanced ribosome collisions, revealing a depletion in cellular RQC activity. Taken together, our data are consistent with a model in which vaccinia virus-mediated reprogramming of host translation results in ribosome collisions that must be cleared to recycle ribosomes back into the available pool to maintain high vaccinia virus mRNA translation activity required for optimal viral production.

## DISCUSSION

Viruses are entirely dependent on the host for translation of their proteins. Indeed, several mechanisms allow viruses to efficiently hijack cellular machinery for optimal viral translation initiation, elongation and termination (Jan et al., 2016). However, several different host mechanisms have also evolved to limit viral protein translation, including PKR-mediated eIF2α phosphorylation as a means to prevent viral mRNA translation initiation (Stern-Ginossar et al., 2019). These viral and host adaptations reveal the importance of translation control for the ongoing conflict between viruses and the hosts they infect.

Less well studied is the role that RQC factors may play in viral infection and host control of viral infections. Recent discoveries have revealed the important regulatory role RQC plays during host translation, especially under conditions that elevate the frequency of ribosome collisions (Joazeiro, 2019). A large number of evolutionarily conserved proteins coordinate RQC within a signaling hierarchy (Sitron and Brandman, 2020). The physiological role for RQC factors in maintaining ribosome availability is exemplified by Pelota, which assists in recycling stalled ribosomal subunits (Guydosh and Green, 2014; Pisareva et al., 2011), and where loss-of-function results in diminished translation of globin mRNAs in blood cells (Mills et al., 2016). Despite these studies, the physiological relevance of many RQC components remains uncharacterized. Of particular interest is the E3 ubiquitin ligase ZNF598, which ubiquitylates 40S ribosomal proteins and recruits downstream ribosome rescue factors such as the RNA helicase ASCC3 (Hashimoto et al., 2020; Juszkiewicz et al., 2020b; Matsuo et al., 2017). The important role that RQC plays in resolving collided ribosomes and other stalled ribosomes during proteotoxic stress suggests that these factors may become particularly important during viral infection. Indeed, while ribosomes are among the most abundant cellular machines in nearly all cells, RQC factors are several-fold less abundant than ribosomes (Itzhak et al., 2016), suggesting that a large burden on the RQC system could possibly overwhelm its capacity.

To address the physiological importance of RQC activity, we investigated its role during viral infection and the host response. We focused on vaccinia virus, as this virus is known to substantially redistribute ribosomes for selective translation of viral mRNAs and to prevent PKR-mediated eIF2α phosphorylation. Consistent with increased translation stress, our work shows that RQC is activated during vaccinia virus infection. Similar to virus-induced proteotoxic stress, we observed increased translation stress upon exposure to poly(I:C) or UV that is revealed by disabling eIF2α phosphorylation. These data led us to test whether RQC may become limiting during vaccinia virus infection by disrupting ZNF598 or one of its primary ubiquitylation targets on the 40S ribosomal subunit, uS10. Consistent with previous work (DiGiuseppe et al., 2018), we found that ZNF598 and its uS10 target are required for optimal vaccinia virus replication. Here, we demonstrated that chronic loss of RQC activity neither results in constitutive activation of inflammatory signaling, nor impairs the transcriptional response to interferon stimulation or vaccinia virus infection. By performing quantitative proteomic and RNA-seq analyses after vaccinia virus infection, we have found that there is a strong posttranscriptional blockade to protein synthesis of nearly every viral protein measured, whereas the host proteome is left relatively unchanged. Our data suggest that there is an early kinetic delay in viral protein production in RQC-deficient cells that likely first impacts early protein synthesis and is further exacerbated when late protein production becomes evident. These results are thus consistent with an increased reliance on RQC pathways during vaccinia virus infection to clear collided ribosomes that result from the dual insults of increased proteotoxic stress and antagonism of eIF2α-mediated translation arrest.

**Fig. 7.**
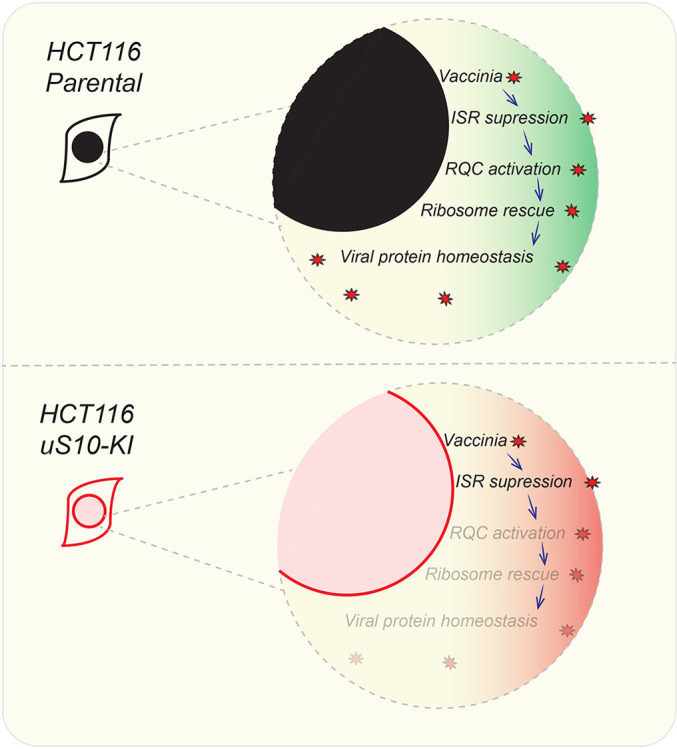
**Model of RQC activation and ribosome depletion during vaccinia virus replication.** In the upper panel, vaccinia virus infection in parental cells and subsequent ISR suppression results in RQC activation and ribosome rescue with normal viral protein synthesis. In the lower panel, ISR suppression by vaccinia virus in RQC-defective cells induces futile ribosome collisions and loss of ribosome rescue.

Based on our data, we propose the following model for how RQC is impacted and plays a functional role during vaccinia virus infection (Fig. 7). Vaccinia virus infection results in both an increased burden on the translation machinery, as well as a suppression of the cellular ISR through antagonism of eIF2α phosphorylation. Both of these consequences of vaccinia virus infection aggravate ribosome collisions. In wild-type cells, RQC activation upon ribosome collisions rescues collided ribosomes, allowing re-entry of these ribosomes into the pool of ribosomes available for active translation. This replenished pool of ribosomes allows optimal viral protein synthesis. However, in RQC-deficient cells, either through loss of ZNF598 or mutation of the primary ubiquitylation target on uS10, vaccinia virus-induced ISR suppression and ribosome collisions are no longer resolved by the RQC pathway. As a result, a cycle of dysfunctional ribosome collisions leads to a depleted ribosome pool and a decrease in viral protein synthesis.

Our work indicates that the evolution of a viral response to the host ISR has exposed the virus to a conditional requirement for the RQC pathway in regulating translational reprogramming. While ribosome collisions are part of the regular translation cycle, feedback inhibition of translation initiation through the ISR normally curtails overt RQC activation (Hickey et al., 2020; Juszkiewicz et al., 2020a; Sinha et al., 2020; Vind et al., 2020a; Wu et al., 2020). When viruses interfere with this feedback inhibition, such as vaccinia virus antagonism of PKR-mediated eIF2α phosphorylation, non-productive translation cycles ensue and therefore require RQC-based ribosomal rescue. Although RQC deficiency does not impact HSV-1 or VSV replication (DiGiuseppe et al., 2018), it is possible that other viruses that alter ISR responses are conditionally dependent on the RQC pathway for propagation.

## MATERIALS AND METHODS

### Cell lines

HCT116 (ATCC), HEK293T (ATCC) and MEF (wild-type and S51A) cells (Scheuner et al., 2001) were grown in DMEM (high glucose, pyruvate and L-glutamine; GIBCO) medium supplemented with 10% FBS (Omega Scientific) and were maintained in humidified incubator supplied with 5% CO_2_ atmosphere. ZNF598 KO cells were validated and characterized previously (Sundaramoorthy et al., 2017). uS10 knock-in (uS10-KI; K4R/K8R) and eS10 knock-in (eS10-KI; K138R/K139R) HCT116 cells were generated and characterized previously (Garshott et al., 2020). Cells were tested for mycoplasma contamination biweekly using MycoAlert (Lonza).

### Reagents and antibodies

The following chemical reagents were used at the indicated concentrations: ISRIB (Tocris), 200 nM; dithiothreitol (DTT; Sigma-Aldrich), 5 mM; and poly(I:C), 15 μg (Invivogen). Cells were irradiated using a Spectrolink UV crosslinker at energy setting 200 J/m^2^. The following primary antibodies were used: rabbit polyclonal anti-RPS10/eS10 (1:2000; Abclonal, A6056), rabbit polyclonal anti-ISG15 (1:5000; Cell Signaling Technology, 2743), rabbit polyclonal anti-ZNF598 (1:7000; Sigma-Aldrich, HPA041760), rabbit monoclonal anti-RPS20/uS10 (1:1000; Abcam, ab133776), rabbit monoclonal anti-phospho-eIF2α (Ser51) (1:2000; Cell Signaling Technology, D9G8) and mouse monoclonal anti-α-tubulin (1:10,000; Cell Signaling Technology, DM1A). The following secondary antibodies were used (at 1:5000 dilution): HRP-conjugated anti-rabbit IgG (H+L) (Promega, W4011) and HRP-conjugated anti-mouse IgG (H+L) (Promega, W4021). Cells were transfected using Lipofectamine RNAiMax (Thermo Fisher Scientific) for poly(I:C) transfections and with Lipofectamine 2000 (Thermo Fisher Scientific) for plasmid transfections. Cells were treated with indicated units of interferon-β for 16 h.

### Poly(A) luciferase plasmids

Firefly luciferase with an N-terminal HA affinity tag (Addgene) was inserted with tandem poly(A) runs using the following forward primers and stop codon-containing reverse primer via the Gateway cloning system (Thermo Fisher Scientific): 4A_FLUC_BP_FOR, 5′-GGGGACAACTTTGTACAAAAAAGTTGGC**AAAA**ATGTACCCATACGATG-3′; 8A_FLUC_BP_FOR, 5′-GGGGACAACTTTGTACAAAAAAGTTGGC**AAAAAAAA**ATGTACCCATACGATG-3′; 12A_FLUC_BP_FOR, 5′-GGGGACAACTTTGTACAAAAAAGTTGGC**AAAAAAAAAAAA**ATGTACCCATACGATG-3′; 20A_FLUC_BP_FOR, 5′-GGGGACAACTTTGTACAAAAAAGTTGGC**AAAAAAAAAAAAAAAAAAAA**ATGTACCCATACGATG-3′; and FLUC_BP_STOP_REV, 5′-GGGGACAACTTTGTACAAGAAAGTTGGctacacggcgatctttccgcc-3′. Poly(A) runs are shown in bold, BP recombination sites are shown in lowercase.

### Luciferase assay

Cells were transfected with 1:1 ratio of plasmids encoding firefly luciferase with varied poly(A) sequence lengths within the 5′ UTR and a control Renilla luciferase plasmid (Addgene) using Lipofectamine 2000. Luciferase activity was measured using a Dual-Glo reagent kit (Promega) and a GloMax (Promega) luminometer, following the manufacturer’s instructions.

### Virus infection and plaque assays

Vaccinia virus strain WR was a generous gift from Richard Condit (University of Florida, FL). For viral titering assays, cells were seeded in 24-well plates and grown overnight, followed by addition of 10,000 plaque-forming units (PFU)/well vaccinia virus. At 24 h after infection, resulting vaccinia virus was harvested by freeze-thaw lysis of infected cells. The resulting supernatant was serially 10-fold diluted in 24-well plates in DMEM containing 10% FBS and overlaid on BSC40 cells (ATCC) at 80% confluency. After 48 h, the medium was aspirated, and the cells were stained with 0.1% Crystal Violet in 20% ethanol, and then de-stained with 20% ethanol. Initial viral concentrations were determined by manually counting plaques. For proteomic, RNA-seq and western blotting analyses, vaccinia virus was added at an MOI of 5 and incubated for the indicated time before cells were harvested.

### RQC FACS assay

Cells were transfected with equal concentration of the dual GFP–ChFP fluorescence reporter containing K20 internal repeat [poly(A)] or K0 control (Sundaramoorthy et al., 2017) in 293T cells. Fluorescence levels were measured using an X-20 Fortessa (BD) instrument, as described previously (Sundaramoorthy et al., 2017).

### RNA-seq

Total RNA from mock-treated or vaccinia-infected cell lines was extracted using Trizol reagent (Invitrogen). Illumina Stranded TruSeq total RNA kit was used for library preparation. Total RNA depleted of ribosomal RNA using Ribo-Zero was converted to cDNA, and the resulting single-read 75 bp library was sequenced on an Illumina Hi-Seq instrument. The demultiplexed and de-indexed FASTQ files were processed using the Galaxy platform (https://usegalaxy.org/; Afgan et al., 2018). Briefly, FASTQ files were quality-control checked using FASTQC (https://www.bioinformatics.babraham.ac.uk/projects/fastqc/) and aligned to the human genome (Hg38) using HISAT2 (Kim et al., 2015). The aligned BAM files were used for counting gene features using FEATURECOUNTS (Liao et al., 2014). The DEBROWSER package (Kucukural et al., 2019) was used for differential gene expression analysis using DESEQ2 (Love et al., 2014) and visualization of volcano plots and heatmaps. FUNRICH (Pathan et al., 2015) was used for generating gene set Venn diagrams and to identify overlapping gene sets. To map reads to the vaccinia genome, the FASTQ files were aligned with custom indexed vaccina WR genome (AY243312.1) using BWA (Li and Durbin, 2009). HTSeq counts (Anders et al., 2015) was used to identify mapped reads with genomic features. DEBROWSER was used as described above for identifying differentially expressed vaccinia genes. Heatmaps and scatterplots were generated using Graphpad Prism (9.0.0). Gene ontology analysis was performed using Funrich (Fonseka et al., 2020).

### Immunoblotting

Total cell lysates were prepared as follows. Cells after treatment were washed with phosphate-buffered saline (PBS) and trypsinized, pelleted and stored at −80°C. Frozen cell pellets were lysed using urea lysis buffer [8 M urea, 50 mM Tris-HCl (pH 8.0), 75 mM NaCl, 1 mM NaF, 1 mM NaV, 1 mM β-glycerophosphate and 25 mM NEM]. Lysates were quantified using a BCA assay (Pierce). Total protein (25 μg) was resolved on 4–20% Tris-glycine SDS–PAGE gels (Bio-Rad). Resolved proteins were blotted to PVDF membranes (Bio-Rad Immuno-Blot) using Bjerrum semi-dry transfer buffer [48 mM Tris, 39 mM glycine, 0.0375% (w/v) SDS, 20% (v/v) methanol, pH 9.2) in a Bio-Rad Trans-Blot Turbo transfer apparatus at 30V constant for 30 mins. Blots were incubated with relevant primary and HRP-conjugated secondary antibodies and were detected using Clarity ECL reagent (Bio-Rad) on a Bio-Rad ChemiDoc XRS+ system.

### SILAC labeling and mass spectrometry

Cells were grown in a medium containing either light (K0) lysine or ^13^C_6_^15^N_2_-labeled (K8) lysine (Cambridge Isotopes) and were processed for mass spectrometry as described previously (Markmiller et al., 2019). Briefly, cells were lysed using 8 M urea lysis buffer, and lysates were quantified for protein content using a BCA assay. 20 µg of total cell extracts each from heavy and light SILAC-labeled samples were combined and diluted to a final urea concentration of 1 M and then digested overnight with LysC enzyme (Promega) at a 1:100 (enzyme:protein) ratio. The digests were reduced with 1 mM DTT for 30 min and then alkylated with 5 mM iodoacetamide (IAA) in the dark for 30 min. The digests were desalted using the Stage Tip method and analyzed by liquid chromatography-tandem mass spectrometry (LC-MS/MS), as described previously (Markmiller et al., 2019). The resultant RAW files were analyzed using Andromeda/MaxQuant (version 1.6.12.0) using the combined UniProt reviewed-only database for *Homo sapiens* (Dec 2020) and UniProt reviewed database for vaccinia virus WR (Dec 2020). The default Andromeda/MaxQuant parameters were used, except the enzyme was selected as LysC and ‘match between the runs’ and ‘requantify’ options were enabled in the MaxQuant settings. The multiplicity criteria for the SILAC label was set as 2.

## Supplementary Material

Supplementary information

Reviewer comments
