# Supplementary material for: Ribosome quality control activity potentiates vaccinia virus protein synthesis during infection
Source: J Cell Sci. 2021 Apr 28;134(8):jcs257188. doi: 10.1242/jcs.257188 (PMC8106952; doi:10.1242/jcs.257188)
Supplement: Supplementary information [file joces-134-257188-s1.pdf]

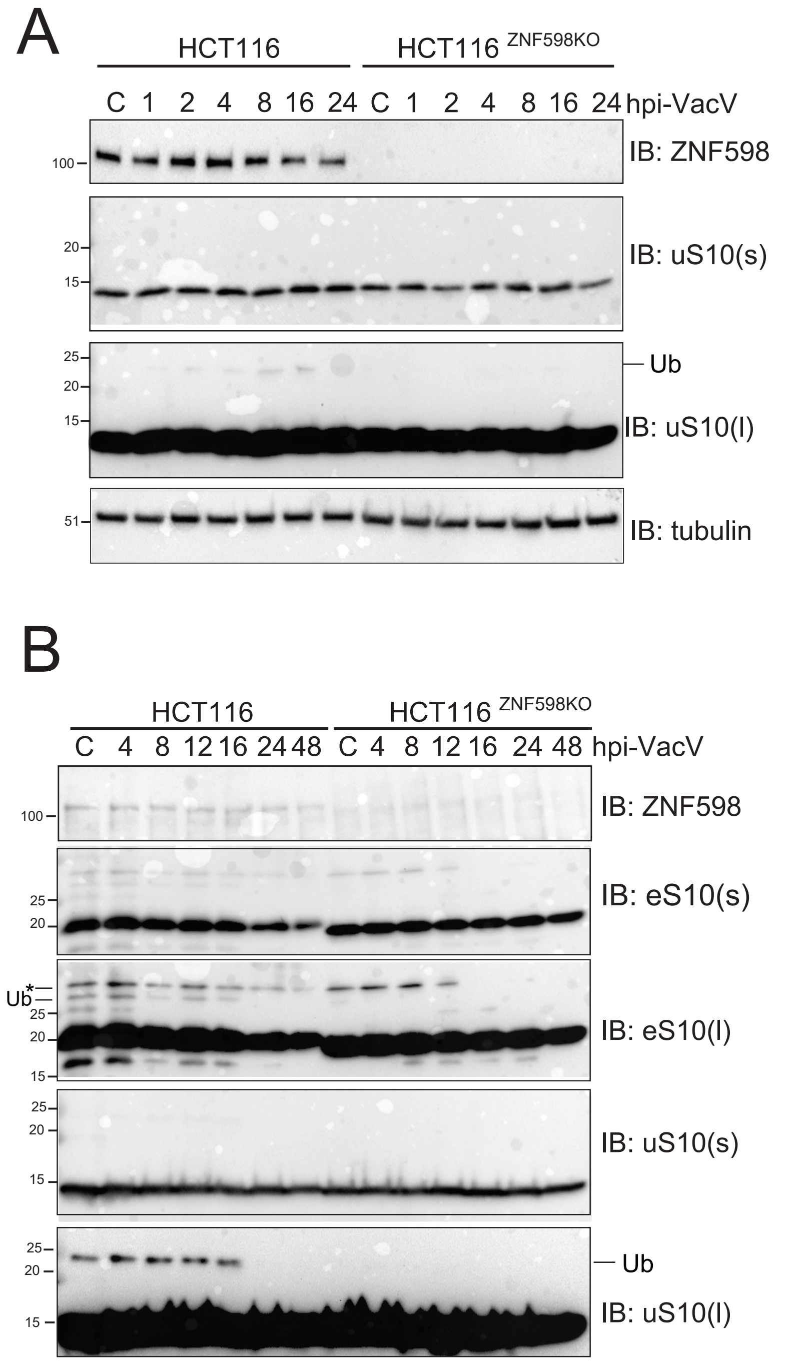

Figure S1

A,B) Parental or ZNF598 knockout (KO) HCT116 cells were mock infected (C) or infected with VacV (MOI=5) and cells were collected at the indicated time points and analyzed by SDS-PAGE and immunoblotted with indicated antibodies.

Journal of Cell Science • Supplementary information

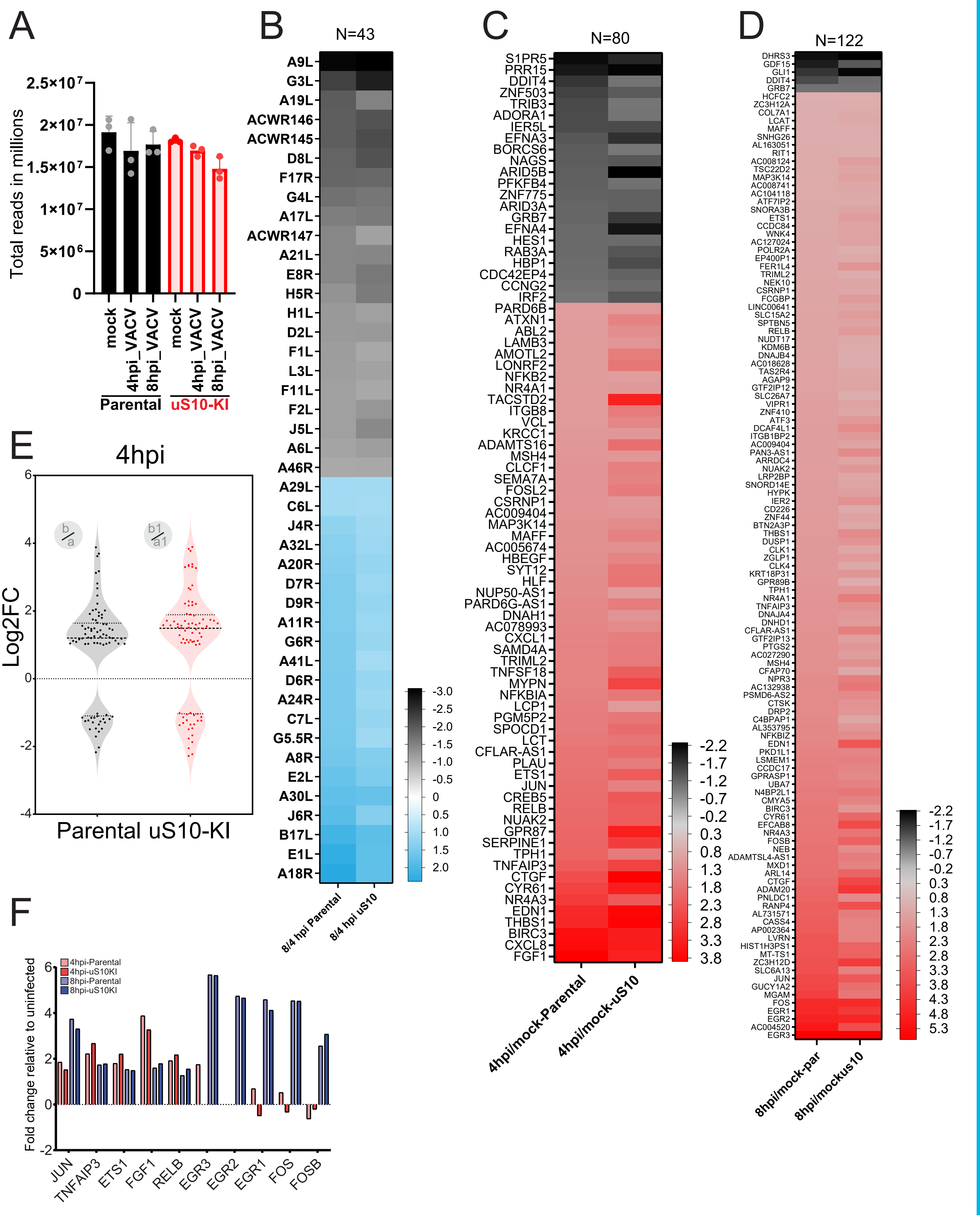

Journal of Cell Science • Supplementary information

Figure S2

A) Bar-graph depicting the mean RNA-seq total reads in each indicated timepoints. Error bars denote SD of triplicate samples.

B) Heat map depicting 43 differentially expressed (DE) vaccinia genes that overlap between the respective 8hpi contrasted with 4hpi within the same cell line (Table S5, 6). DE genes compared between parental and uS10-KI cell lines. Gene names are indicated on the left. Upregulated genes are represented in blue and downregulated in black. The log_2 fold change range is between +2.3 to -3.

C) Heat map depicting 80 differentially expressed human genes that overlap between parental and uS10-KI cell lines when comparing mock infected cells with those 4 hours after vaccinia virus infection (Table S12, 13). Upregulated genes are represented in red and downregulated in black. The log_2 fold change range is between +3.8 to -2.2.

D) Heat map depicting 122 differentially expressed human genes that overlap between parental and uS10-KI cell lines when comparing mock infected cells with those 8 hours after vaccinia virus infection (Table S10, 11). Upregulated genes are represented in red and downregulated in black. The log_2 fold change range is between +5.6 to -2.2.

E) Violin plot showing frequency distribution of the log_2 ratios of the 80 common differentially expressed host genes comparing uninfected cells to those 4 hours post infection (hpi) (depicted in C) within the indicated cell lines (Table S12, 13).

F) Fold change of the average mean feature count for triplicate samples for the indicated transcripts relative to uninfected control for parental (light shades) and uS10-KI (dark shades) cells at 4 (red) and 8 (blue) hours post infection.

Table S1: List of SILAC labelled proteins identified in ZNF598 Knockout cells during Vaccinia infection time course (both Human and Vaccinia).

Click here to download Table S1

Table S2: List of SILAC labelled proteins identified in ZNF598 Knockout cells during Vaccinia infection time course (Vaccinia).

Click here to download Table S2

Table S3: List of SILAC labelled proteins identified in uS10-KI knock-in cells during Vaccinia infection time course (both Human and Vaccinia)

Click here to download Table S3

Table S4 List of SILAC labelled proteins identified in uS10-KI knock-in cells during Vaccinia infection time course (Vaccinia).

Click here to download Table S4

Table S5: List of differentially expressed Vaccinia genes in HCT116- Parental cells comparing 8 hours post infection (hpi) with 4hpi from the RNASeq experiment.

Click here to download Table S5

Table S6: List of differentially expressed Vaccinia genes in HCT116- uS10-KI cells comparing 8 hpi with 4hpi from the RNASeq experiment.

Click here to download Table S6

Table S7: List of differentially expressed Vaccinia genes between HCT116- Parental cells and HCT116-uS10-KI cells at 4hpi from the RNASeq experiment.

Click here to download Table S7

Table S8: List of differentially expressed Vaccinia genes between HCT116- Parental cells and HCT116-uS10-KI cells at 8hpi from the RNASeq experiment.

Click here to download Table S8

Journal of Cell Science • Supplementary information

Table S9: Consolidated list of Vaccinia proteins and transcripts from SILAC and RNASeq datasets in ZNF598 knockout (SILAC alone) and Us10-KI knock-in cells (Both SILAC and RNASeq).

Click here to download Table S9

Table S10: List of differentially expressed Human genes in HCT116-Parental cells comparing 8hpi with mock treated cells from the RNASeq experiment.

Click here to download Table S10

Table S11: List of differentially expressed Human genes in HCT116-uS10-KI cells comparing 8hpi with mock treated cells from the RNASeq experiment.

Click here to download Table S11

Table S12: List of differentially expressed Human genes in HCT116-Parental cells comparing 4hpi with mock treated cells from the RNASeq experiment.

Click here to download Table S12

Table S13: List of differentially expressed Human genes in HCT116-uS10-KI cells comparing 4hpi with mock treated cells from the RNASeq experiment.

Click here to download Table S13

Table S14: List of differentially expressed Human genes in cells at 4hpi with vaccinia, comparing HCT116-Parental with HCT116-uS10-KI.

Click here to download Table S14

Table S15: List of differentially expressed Human genes in cells at 8hpi with vaccinia, comparing HCT116-Parental with HCT116-uS10-KI.

Click here to download Table S15

Table S16: List of differentially expressed Human genes in mock treated cells comparing HCT116- Parental with HCT116-uS10-KI.

Click here to download Table S16
