# Supplementary material for: Ribosome quality control activity potentiates vaccinia virus protein synthesis during infection
Source: J Cell Sci. 2021 Apr 28;134(8):jcs257188. doi: 10.1242/jcs.257188 (PMC8106952; doi:10.1242/jcs.257188)
Supplement: Reviewer comments [file joces-134-257188_review_history.pdf]

Ribosome quality control activity potentiates vaccinia virus protein synthesis during infection

Elayanambi Sundaramoorthy, Andrew P. Ryan, Amit Fulzele, Marilyn Leonard, Matthew D. Daugherty and Eric J. Bennett
DOI: 10.1242/jcs.257188

Editor: Michael Way

Review timeline

Original submission:	16 November 2020
Editorial decision:	14 December 2020
First revision received:	3 February 2021
Editorial decision:	2 March 2021
Second revision received:	11 March 2021
Accepted:	12 March 2021

Original submission

First decision letter

MS ID#: JOCES/2020/257188

MS TITLE: Ribosome quality control activity potentiates vaccinia virus protein synthesis during infection

AUTHORS: Elayanambi Sundaramoorthy, Andrew P Ryan, Amit Fulzele, Marilyn Leonard, Matthew Daugherty, and Eric J Bennett
ARTICLE TYPE: Research Article

We have now reached a decision on the above manuscript.

To see the reviewers' reports and a copy of this decision letter, please go to: https://submit-jcs.biologists.organd click on the 'Manuscripts with Decisions' queue in the Author Area. (Corresponding author only has access to reviews.)

As you will see, the reviewers are positive but raise a number of criticisms that prevent me from accepting the paper at this stage. They suggest, however, that a revised version might prove acceptable, if you can address their concerns, many of which involve re-examination of the data and a change in presentation. On the whole, I think the reviewers questions are constructive and will improve the accessibility of your study to the JCS readership. If you think that you can deal satisfactorily with the criticisms on revision, I would be pleased to see a revised manuscript. We would then return it to the reviewers.

We are aware that you may be experiencing disruption to the normal running of your lab that makes experimental revisions challenging. If it would be helpful, we encourage you to contact us to discuss your revision in greater detail. Please send us a point-by-point response indicating where you are able to address concerns raised (either experimentally or by changes to the text) and where you will not be able to do so within the normal timeframe of a revision. We will then provide further guidance. Please also note that we are happy to extend revision timeframes as necessary.

© 2021. Published by The Company of Biologists under the terms of the Creative Commons Attribution License (https://creativecommons.org/licenses/by/4.0/).

Please ensure that you clearly highlight all changes made in the revised manuscript. Please avoid using 'Tracked changes' in Word files as these are lost in PDF conversion.

I should be grateful if you would also provide a point-by-point response detailing how you have dealt with the points raised by the reviewers in the 'Response to Reviewers' box. Please attend to all of the reviewers' comments. If you do not agree with any of their criticisms or suggestions please explain clearly why this is so.

Reviewer 1

Advance summary and potential significance to field

The ribosome-associated quality control (RQC) pathway is a quality control system that identifies elongating ribosomes whose progression is stalled the translating mRNA, which recognizes and catalyzes the mRNA and nascent polypeptide degradation to allow ribosome subunit recycling for translation of other mRNAs. Vaccinia virus(VACV) is a large DNA virus encoding over 200 proteins. The infection of VACV has an acute and rapid demand for viral protein synthesis using host cell translation machinery. It is of great importance to understand how VACV infection reprograms cellular translation machinery to meet this demand, which is critical in understanding viral replication strategy and mRNA translation in general. In this manuscript entitled "Ribosome quality control activity potentiates vaccinia virus protein synthesis during infection," Sundaramoorthy et al. utilized VACV to investigate the interaction between the RQC pathway and viral protein synthesis. The data shows that ZNF598, an RQC sensor, and uS10 ubiquitylation, a marker of ribosome collision during RQC, are required for optimal VACV replication. VACV infection also reduced overall cellular RQC activity. The authors carried out mass-spec and RNA-Seq to analyze protein and RNA abundance and suggest that loss of ZNF598 function or dysfunction of uS10 resulted in reduced viral mRNA translation. The study highlights an essential aspect of virus-host cell interaction at the protein synthesis interface exemplifying the physiological importance of RQC activity. While a previous paper had reported that ZNF598 is important for the translation of VACV mRNAs with a 5'-poly(A) leader, this manuscript stated that ZNF598 is important for VACV mRNA translation but does not need a 5'-poly(A) leader.

Comments for the author

While this manuscript addresses an important question and is well-written, some conclusions are not sufficiently supported with the data and analyses, mainly the conclusion that RQC pathway dysfunction suppresses VACV mRNA translation. This reviewer raises the following points for the authors' consideration to improve the manuscript.

1. Fig. 4AB, the authors stated that "Direct comparison of viral protein abundance with and without reported polyA sequences within the 5'UTR of corresponding mRNAs(DiGiuseppe et al., 2018) did not reveal any difference in either ZNF598 KO or uS10-KI cells." All VACV post-replicative mRNAs have a 5'-poly(A) leader. Those VACV mRNAs without a poly(A) leader are transcribed from early viral genes. If viral early protein synthesis is suppressed, this will likely sequentially suppress viral DNA replication, as well as viral post-replicative mRNA transcription following viral DNA replication. However, in Fig. 5, the authors stated that "our analysis indicates that while a small number of vaccinia virus genes show reduced mRNA levels at 8 hours post-infection, there is no evidence of widespread alternations in vaccinia virus gene transcription in uS10-KI cells (Figure S1B, Table S2)." These results and interpretation are not clear and inconsistent with the cascade expression of VACV genes. This reviewer suggests the following analyses and experiments to clarify this aspect. (1) A detailed and separated analysis of VACV early/intermediate/late mRNA and protein levels of the RNA-Seq/Mass-spec data. The classification of VACV viral early/intermediate/late genes and their poly(A) leader status can be found in the following papers: PMID: 20534518, PMID: 21795349, PMID: 21490097 PMID: 22829601. (2) Pick one to two representative early, intermediate, and late genes, and examine their mRNA and protein levels by qRT-PCR (or Northern blot) and Western Blot, respectively. (3) using an RNA-based reporter system to investigate the translation of RNA with or without a poly(A) head in addition to the plasmid-based reporter system. The interpretation of the plasmid-based reporter system is complicated by plasmids that can replicate in VACV-infected

© 2021. Published by The Company of Biologists under the terms of the Creative Commons Attribution License (https://creativecommons.org/licenses/by/4.0/).

mammalian cells (PMID: 15784143) and transcription regulation in addition to translational regulation.

2. The authors stated that “Consistent with the viral titer data, vaccinia virus infection resulted in enhanced uS10 ubiquitylation throughout the infection time course indicating that vaccinia virus infection induces ribosome collisions during cellular replication” Fig. 2C actually doesn’t show notable uS10 ubiquitylation at 4-16 hpi, and no uS10 ubiquitylation observed at 24 and 48 hpi.

3. Does ZNF598 KO or uS10 mutation affect cell viability that may impact VACV replication?

4. Statistical analyses were not provided for some of the figures, e.g., Fig. 2AB

5. The authors stated that "However, contrary to previous reports suggesting that translation of late vaccinia virus mRNAs containing non-templated polyA sequences of various lengths within their 5’UTR were specifically impacted by a loss of ZNF598 activity, we demonstrate that viral protein production is broadly repressed in ZNF598 KO or uS10-KI cells.” A more thorough discussion of potential reasons for the discrepancy would benefit the readers.

<u>Reviewer 2</u>

Advance summary and potential significance to field

In this manuscript, Sundaramoorthy and colleagues investigate the role of RQC factors in vaccinia virus (VacV) infection using high throughput “omics” approaches. Overall, the findings provide important advances and new understanding in the field. That said, the report is effectively in two parts that each have issues that need to be addressed. The first part largely confirms prior studies, which is important in itself, but some of the conclusions are not supported by the data and are confusingly presented. Some of the proposed mechanisms seem counter to the actual data and there would seem to be simpler explanations that make the findings no less important. The second part, and the greatest advance in this study, is the broader proteomic and genomic analyses. This has the power to be incredibly informative but it is presented in a generalized non-identifiable manner that to me, doesn’t exploit the full power of the data in hand and as is, just leaves one with a generic overview and pondering more questions than answers. Addressing this seems like easy dry-lab work that is ideally suited to revisions in the current Covid climate and would make this study quite valuable to the field.

Comments for the author

Major Concerns:
The true novelty and power of this report lies in the broad “omic” analyses but the actual important information is generalized and lost to the reader in the form of unidentifiable data points presented in graphs.
Obviously the information is in the raw data excel sheets but they are impossible to sift through and I think the data should be presented in a more directly informative way that would change this from a descriptive data mashup into something that is truly valuable.

In all of the proteomic data each viral protein is presented as an unidentified data point for statistical analysis. This makes it hard to tell if all proteins behave the same and there is a uniform response, or if each protein behaves differently and there is complexity in how RQC affects infection. By way of simple example, in figure 3B and 3C at 8h there are some proteins that are higher in ZNF KO cells than parental – what are these proteins? They are ignored in favor of the general downward trend. Overall, “data points” often lie in a similar region but one cannot tell if these are proteins that have dropped down from a higher expression level, risen up or stay the same because each protein circle has no identity. It would be very helpful to generate supplemental figures where each protein is presented in parental vs KO/KI side by side so we can get a sense of what is actually happening. It would also be helpful to assign whether the proteins are early, intermediate or late as it aids kinetic understanding.

© 2021. Published by The Company of Biologists under the terms of the Creative Commons Attribution License (https://creativecommons.org/licenses/by/4.0/).

There is a similar issue with the transcript analysis, which is also only done at 4h and 8h while the bulk of significant changes in protein levels occurs at later stages. While I don't think there is a need to go and do the RNA analysis at these later timepoints, especially during Covid, the data could be framed to increase its relevance. For example, it seems 200 transcripts are analyzed at each timepoint yet far fewer proteins are detected by proteomics. Perhaps an additional figure could be generated that matches the transcript and protein, in an identifiable manner, to get some sense of whether specific mRNA changes relate to specific protein changes. Again, this study has the potential to be very powerful but the generalization of the data greatly weakens this.

How proteins were assigned polyA or non-polyA status is not described in the results or methods, and raises concerns. There is a simple reference to DiGiuseppe, 2018 but this paper only analyses short polyA tracts in the coding sequence of VacV transcripts, not the 5' polyA leaders. Yang and Moss performed 5' leader analysis. Although reporter assays suggest RQC factors don't specifically regulate 5 polyA translation, this is important to clarify in the proteomic analysis both in terms of the data itself and the methods as to how assignments were done. It would also be informative to add estimated leader lengths from the Yang analysis if possible as there may be correlative or lack of correlative evidence to support the reporter assays.

Data suggesting that infection induces uS10 or eS10 ubiquitylation in figure 2C is really not convincing. There seems to be no change and this needs careful quantification relative to endogenous forms, and seems likely to be extremely modest at best. The findings are also in line with prior reports, so why interpret the data otherwise?

In figure 1, the most robust uS10/eS10 ubiquitylation are seen in MEFs treated with UV (panel B) but polyI:C doesn't do much, levels are just elevated without eIF2 phosphorylation in the S51 mutant (panel A). In figure 1C, human cells seem to have very different ubiquitylated species to the MEF blots, there is no ISRIB alone control to show that like MEFs it's not just eIF2 that is causing this increase, and comparing to Figure 2, the designation of Ub bands seems somewhat arbitrary. Either way, the changes are not very robust or convincing. This is not a fatal flaw as the mutants show these events are functional, but it seems the data is very heavily interpreted in the absence of rigorous quantification for such small changes.

Related to this, a model is evoked that ZNF598 might be sequestered away to explain why infection increases RQC reporter activity. But if the virus is sequestering away ZNF598 then why would ZNF598 KO have any impact? Wouldn't a very simple model that also fits with a lack of uS10/eS10 ubiquitylation in the points above be that RQC capacity is near maximum activity in human cells, given that it is relatively low abundance relative to ribosomes anyway, and that the virus simply overloads the cell's RQC capacity resulting in reporter plasmids now showing readthrough activity because ZNF598 can't get to them anymore?

Minor Concerns
In figure 5 there are 332 and 228 differentially regulated host genes between parental and uS10-KI, but only 122 overlap. Does this mean there are actually substantial differences in host responses? This could actually be quite interesting to point out, more interesting than a smaller set of common genes?

It would be good to clarify eS vs RPS nomenclature for general readers especially since uS10 is RPS20, which is confusing to non-experts.

It would be nice to acknowledge Wang et al, Cell Rep, 2019, who also showed ZNF598 regulates ISGs through RIG-I. The results shown for IFN are in line with this and prior studies, and the fact that IFN can induce ISGs in these cells isn't surprising as it's unrelated to the ZNF598 mechanism reported previously. The relevance of this finding could perhaps be better framed against prior studies.

© 2021. Published by The Company of Biologists under the terms of the Creative Commons Attribution License (https://creativecommons.org/licenses/by/4.0/).

First revision

Author response to reviewers' comments

Reviewer #1
Fig. 4AB, the authors stated that "Direct comparison of viral protein abundance with and without reported polyA sequences within the 5'UTR of corresponding mRNAs(DiGiuseppe et al., 2018) did not reveal any difference in either ZNF598 KO or uS10-KI cells." All VACV post-replicative mRNAs have a 5'-poly(A) leader. Those VACV mRNAs without a poly(A) leader are transcribed from early viral genes. If viral early protein synthesis is suppressed, this will likely sequentially suppress viral DNA replication, as well as viral post-replicative mRNA transcription following viral DNA replication. However, in Fig. 5, the authors stated that "our analysis indicates that while a small number of vaccinia virus genes show reduced mRNA levels at 8 hours post- infection, there is no evidence of widespread alternations in vaccinia virus gene transcription in uS10-KI cells (Figure S1B, Table S2)." These results and interpretation are not clear and inconsistent with the cascade expression of VACV genes. This reviewer suggests the following analyses and experiments to clarify this aspect.

(1) A detailed and separated analysis of VACV early/intermediate/late mRNA and protein levels of the RNA-Seq/Mass-spec data. The classification of VACV viral early/intermediate/late genes and their poly(A) leader status can be found in the following papers: PMID: 20534518, PMID: 21795349, PMID: 21490097, PMID: 22829601.
We thank the reviewer for this suggestion and have reanalyzed both the proteomics and RNA- seq data by breaking the VacV genes/proteins into early vs intermediate/late (post-replicative) categories to visualize any differences between these temporal groups. This data is now represented in Fig 4A-D and Fig 5C,D. We have also now included this temporal gene expression classification in tables S1-S9. This analysis revealed no significant differences in protein or mRNA expression between early and post-replicative VacV genes which suggests a much broader mechanism of VacV protein repression in RQC deficient cell lines. We have also made a consolidated excel table within table S9 with all detected VacV genes and/or proteins from our RNA-seq and proteomics data. This makes it easier to directly compare RNA-seq and proteomics data across the VACV genes as well as columns that indicate their temporal expression pattern.

(2) Pick one to two representative early, intermediate, and late genes, and examine their mRNA and protein levels by qRT-PCR (or Northern blot) and Western Blot, respectively.

While we appreciate the suggestion, we feel that our quantitative proteomics data need not be validated using often unverified antibodies against VacV proteins. Further, we would have to obtain these reagents from either commercial sources (again whose reliability is often unknown) or from other researchers (if they are willing to share these reagents). Due to current experimental limitations in the lab we feel these experiments would not add substantially to the current manuscript.

(3) using an RNA-based reporter system to investigate the translation of RNA with or without a poly(A) head in addition to the plasmid-based reporter system. The interpretation of the plasmid-based reporter system is complicated by plasmids that can replicate in VACV-infected mammalian cells (PMID: 15784143) and transcription regulation in addition to translational regulation.

We thank the reviewer for this suggestion and were aware of possible issues with plasmid replication during VacV-infected cells. The reviewer is referring to data represented in Figure 4E in which we document that VacV-infected cells containing reporter plasmids with a long poly(A) sequence in the 5'UTR produce more firefly luciferase reporter compared to uninfected cells. While we see the value in performing a similar experiment using mRNA transcribed from this plasmid, our data is completely consistent with numerous other studies documenting that VacV- infected cells display an enhanced ability to translate mRNAs with 5'UTR polyA sequences via characterized mechanisms. Combined with our limited ability to perform wet-lab experiments at this time due to COVID-related restrictions, we feel that this additional experiment would merely confirm a well-established finding. We also note that all additional experiments showing cells with defective RQC responses show no difference in translating 5'UTR poly(A) reporters compared to control cells is done in the absence of VacV infection and would not suffer from this plasmid replication issue.

© 2021. Published by The Company of Biologists under the terms of the Creative Commons Attribution License (https://creativecommons.org/licenses/by/4.0/).

The authors stated that "Consistent with the viral titer data, vaccinia virus infection resulted in enhanced uS10 ubiquitylation throughout the infection time course indicating that vaccinia virus infection induces ribosome collisions during cellular replication" Fig. 2C actually doesn't show notable uS10 ubiquitylation at 4-16 hpi, and no uS10 ubiquitylation observed at 24 and 48 hpi.

We have added additional western blotting data from replicate experiments in HCT116 cells and from 293Flp-IN parental and ZNF598 KO cells indicating that uS10 ubiquitylation is enhanced upon VacV infection. We now include the Flp-IN data in the main manuscript as figure 2C and have also included the blotting data from replicate infections in HCT116 parental and ZNF598 KO cells in supplemental figure 1. We have also quantified the change in uS10 and eS10 ubiquitylation in all three of these experiments and have included that quantification here as reviewer figure 1. This new data, combined with the original data, document a reproducible increase in uS10 ubiquitylation upon VacV infection.

Does ZNF598 KO or uS10 mutation affect cell viability that may impact VACV replication?

There is a slight proliferation decrease observed in the HCT116 ZNF598 KO cells, but not in the uS10 knock-in cells. We have not observed a measurable proliferation defect in the 293Flp-IN ZNF598KO cells which also have a reduced ability to support VACV replication. While the reviewer brings up an excellent point, the observation that there is no consistent decrease in cellular proliferation argues that the VACV-replication defect is not a result of an overall proliferation defect in RQC deficient cells.

Statistical analyses were not provided for some of the figures, e.g., Fig. 2AB

We have added statistical analyses where appropriate and indicated the analysis in the figure legends.

The authors stated that "However, contrary to previous reports suggesting that translation of late vaccinia virus mRNAs containing non-templated polyA sequences of various lengths within their 5'UTR were specifically impacted by a loss of ZNF598 activity, we demonstrate that viral protein production is broadly repressed in ZNF598 KO or uS10-KI cells." A more thorough discussion of potential reasons for the discrepancy would benefit the readers.

We have added some language to the discussion section that addresses this discrepancy.

Reviewer #2

Obviously, the information is in the raw data excel sheets but they are impossible to sift through and I think the data should be presented in a more directly informative way that would change this from a descriptive data mashup into something that is truly valuable.

By way of simple example, in figure 3B and 3C at 8h there are some proteins that are higher in ZNF KO cells than parental – what are these proteins? They are ignored in favor of the general downward trend. Overall, "data points" often lie in a similar region but one cannot tell if these are proteins that have dropped down from a higher expression level, risen up or stay the same because each protein circle has no identity. It would be very helpful to generate supplemental figures where each protein is presented in parental vs KO/KI side by side so we can get a sense of what is actually happening. It would also be helpful to assign whether the proteins are early, intermediate or late as it aids kinetic understanding.

We provided all proteomics data and RNA-seq data as excel spreadsheets to allow readers to search for specific host or VacV genes of interest. Generating individual plots of each VacV or host protein from the combined data would generate a substantial number of supplemental figures that, we feel, would not be as useful as providing the data in a searchable and tabular format. We have reformatted the table containing proteomics and RNA-seq data for VacV genes/proteins to make it easier to directly compare the RNA-seq and proteomics data (see new table S9).

© 2021. Published by The Company of Biologists under the terms of the Creative Commons Attribution License (https://creativecommons.org/licenses/by/4.0/).

There is a similar issue with the transcript analysis, which is also only done at 4h and 8h while the bulk of significant changes in protein levels occurs at later stages. While I don't think there is a need to go and do the RNA analysis at these later timepoints, especially during Covid, the data could be framed to increase its relevance. For example, it seems 200 transcripts are analyzed at each timepoint yet far fewer proteins are detected by proteomics. Perhaps an additional figure could be generated that matches the transcript and protein, in an identifiable manner, to get some sense of whether specific mRNA changes relate to specific protein changes. Again, this study has the potential to be very powerful but the generalization of the data greatly weakens this.

We have made a consolidated table displaying these matched time points for the proteomics and RNAseq datasets in table S9. We have also added a new panel figure 5E that directly compares VacV protein versus transcript fold changes in uS10-KI cells compared to parental cells 8 hours post infection.

How proteins were assigned polyA or non-polyA status is not described in the results or methods, and raises concerns. There is a simple reference to DiGiuseppe, 2018 but this paper only analyses short polyA tracts in the coding sequence of VacV transcripts, not the 5' polyA leaders. Yang and Moss performed 5' leader analysis. Although reporter assays suggest RQC factors don't specifically regulate 5 polyA translation, this is important to clarify in the proteomic analysis both in terms of the data itself and the methods as to how assignments were done. It would also be informative to add estimated leader lengths from the Yang analysis if possible as there may be correlative or lack of correlative evidence to support the reporter assays.

We thank the reviewer for making a similar comment as reviewer#1 and have made additional figures comparing early and intermediate/late VacV genes. We have also added language to the results section to make it more clear which published sources we used to make these classifications.

Data suggesting that infection induces uS10 or eS10 ubiquitylation in figure 2C is really not convincing. There seems to be no change and this needs careful quantification relative to endogenous forms, and seems likely to be extremely modest at best. The findings are also in line with prior reports, so why interpret the data otherwise?

Reviewer #1 made a similar suggestion and we have duplicated our response here.
We have added additional western blotting data from replicate experiments in HCT116 cells and from 293Flp-IN parental and ZNF598 KO cells indicating that uS10 ubiquitylation is enhanced upon VacV infection. We now include the Flp-IN data in the main manuscript as figure 2C and have also included the blotting data from replicate infections in HCT116 parental and ZNF598 KO cells in supplemental figure 1. We have also quantified the change in uS10 and eS10 ubiquitylation in all three of these experiments and have included that quantification here as reviewer figure 1. This new data, combined with the original data, document a reproducible increase in uS10 ubiquitylation upon VacV infection.

In figure 1, the most robust uS10/eS10 ubiquitylation are seen in MEFs treated with UV (panel B) but polyI:C doesn't do much, levels are just elevated without eIF2 phosphorylation in the S51 mutant (panel A). In figure 1C, human cells seem to have very different ubiquitylated species to the MEF blots, there is no ISRIB alone control to show that like MEFs it's not just eIF2 that is causing this increase, and comparing to Figure 2, the designation of Ub bands seems somewhat arbitrary. Either way, the changes are not very robust or convincing. This is not a fatal flaw as the mutants show these events are functional, but it seems the data is very heavily interpreted in the absence of rigorous quantification for such small changes.

We apologize for overstating our interpretation of the data depicted in figure 1. We were merely trying to make the point that when eIF2□ phosphorylation is defective, and translation initiation repression does not occur in response to proteotoxic stress, that this condition leads to elevated ribosome collision events and subsequent ribosome ubiquitylation. The data depicted in figure 1A and 1B show increased uS10 ubiquitylation in the S51A mutant MEFs compared to wild type control MEFs upon polyI:C or UV exposure. While the impact on eS10 is less robust, we feel that these data are consistent with this interpretation and is also in line with published results in yeast (PMID:33338396). We have attempted to clarify this issue in the results section as well as soften our

© 2021. Published by The Company of Biologists under the terms of the Creative Commons Attribution License (https://creativecommons.org/licenses/by/4.0/).

interpretation.

We have also now quantified the ubiquitylated species of uS10 and eS10 in Figure1A-C and have included it here as reviewer figure 2. We are focusing on the following finding from these set of experiments, Suppression of translational initiation is critically dependent on eIF2¤ phosphorylation. Loss in this suppression leads to increased ribosomal collisions as seen by sustained increase in uS10 ubiquitylation levels. Unlike Poly-I:C which is transfected into cells and shows extensive variation, UV treatment induced uniform increase in ubiquitylated species. ISRIB action on translation is eIF2¤ phospho-status dependent and ISRIB under normal conditions does not alter translation. PMID: 25719440

Related to this, a model is evoked that ZNF598 might be sequestered away to explain why infection increases RQC reporter activity. But if the virus is sequestering away ZNF598 then why would ZNF598 KO have any impact? Wouldn't a very simple model that also fits with a lack of uS10/eS10 ubiquitylation in the points above be that RQC capacity is near maximum activity in human cells, given that it is relatively low abundance relative to ribosomes anyway, and that the virus simply overloads the cell's RQC capacity resulting in reporter plasmids now showing readthrough activity because ZNF598 can't get to them anymore?

The reviewer makes an excellent point and we have considered this simpler explanation for the observed VacV-replication defect. We apologize for not making this alternative explanation clearer in the discussion section. We have altered the discussion to more specifically include this interpretation.

In figure 5 there are 332 and 228 differentially regulated host genes between parental and uS10-KI, but only 122 overlap. Does this mean there are actually substantial differences in host responses? This could actually be quite interesting to point out, more interesting than a smaller set of common genes?

The reviewer is correct in noting that there are statistically significant differences to the host transcriptional response in uS10-KI cells upon infection. We now include this analysis in the main text, "While the overwhelming majority of transcripts were altered similarly between parental and uS10-KI cells in response to VacV infection, our analysis revealed 137 and 93 differentially expressed genes comparing parental and uS10-KI cells at 4 and 8 hours post infection, respectively (Table S14, 15,16). We also did differential gene expression comparison of mock treated cells between the two cell lines and identified 112 genes (Table S16). These baseline gene expression differences are unlikely to account for the observed VacV proliferation defect in uS10-KI cells as only one of these genes was found to be differentially expression upon VacV infection in parental cells or uS10 cells. Combined, the RQC deficient uS10-KI cells displayed similar host and viral transcript levels upon vaccinia virus infection. Given the wide fluctuation in viral proteins, we propose that a post-transcriptional mechanism governs the loss of viral protein expression."

We also performed Gene Ontology enrichment analysis of these genes and while this analysis highlighted some differences in pathways involved in general cell biological pathways (signaling, localization, growth) there was no obvious specific cellular pathway which was not activated or repressed in uS10-KI cells vs parental cells. In fact, many of the main transcriptional responses involving RelB, EGR1, Fos, and Jun were induced similarly between uS10 KI and parental cells. We have included this data and a direct correlation plot comparing transcript abundance at the 8 hour time point post infection between parental and uS10-KI cells (Fig. 5F, S2F).

It would be good to clarify eS vs RPS nomenclature for general readers especially since uS10 is RPS20, which is confusing to non-experts.

We agree that the changing ribosome nomenclature generates confusion. To appease both the ribosome field specialists and generalists, we have tried to be more explicit about the nomenclature in the early part of the results and added a succinct nomenclature key to figure 1 to aid comparison between the older (RPS10, RPS20) and updated (eS10, uS10) nomenclature.

It would be nice to acknowledge Wang et al, Cell Rep, 2019, who also showed ZNF598 regulates ISGs through RIG-I. The results shown for IFN are in line with this and prior studies, and the fact

© 2021. Published by The Company of Biologists under the terms of the Creative Commons Attribution License (https://creativecommons.org/licenses/by/4.0/).

that IFN can induce ISGs in these cells isn't surprising as it's unrelated to the ZNF598 mechanism reported previously. The relevance of this finding could perhaps be better framed against prior studies.

Our data demonstrate no overt differences in IFN-induced transcription in our RQC deficient cell lines upon polyI:C or VACV infection. This argues that there is no global alteration in IFN signaling in these cell lines which cannot account for the observed VACV-replication defect. We tried to make this point clear as it runs counter to previous published reports that mostly rely on transient knockdown of ZNF598. It seems clear that there is a substantial difference in IFN signaling when comparing transient ZNF598 knockdown to chronic genetic ablation of ZNF598.

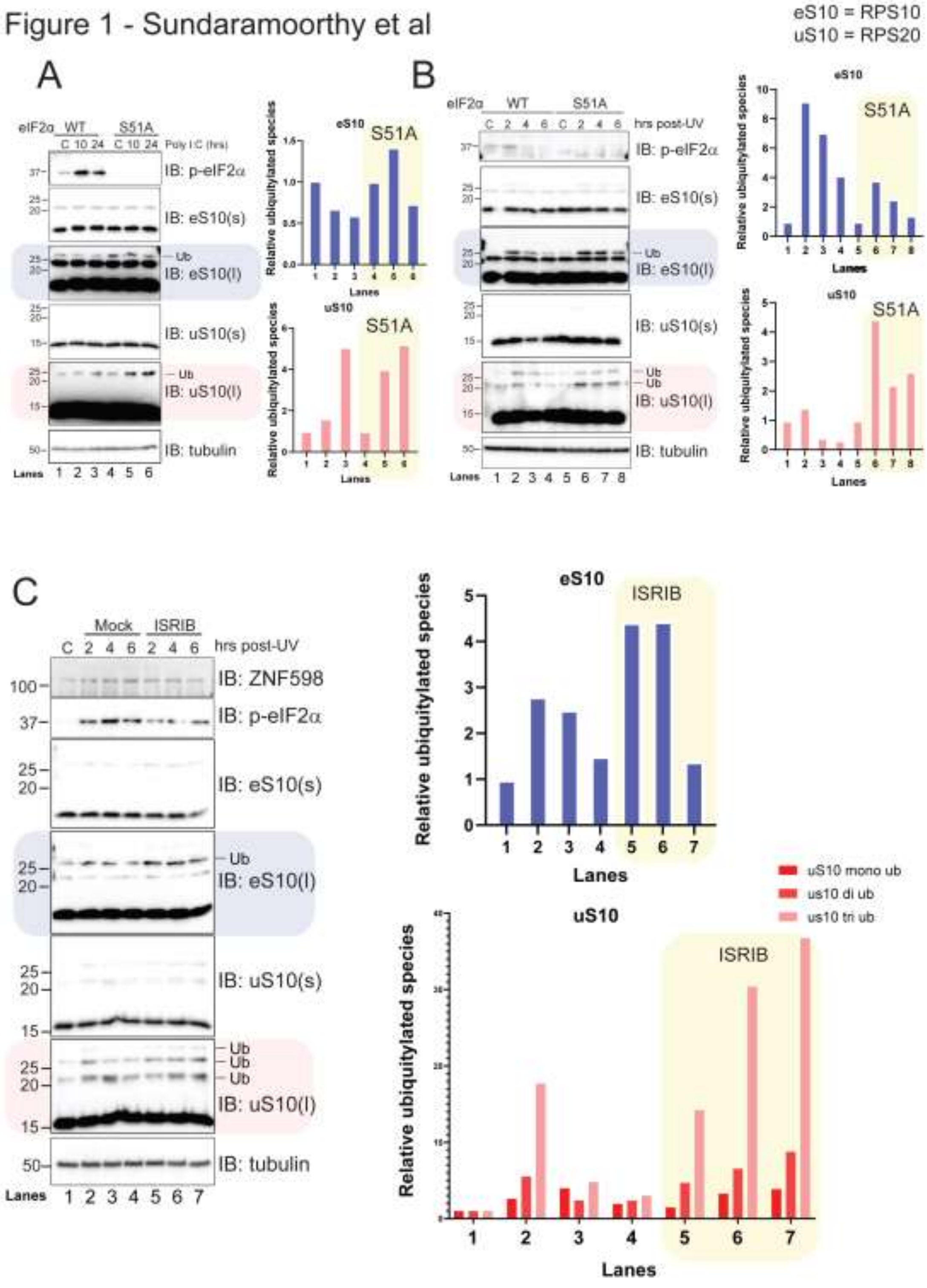

© 2021. Published by The Company of Biologists under the terms of the Creative Commons Attribution License (https://creativecommons.org/licenses/by/4.0/).

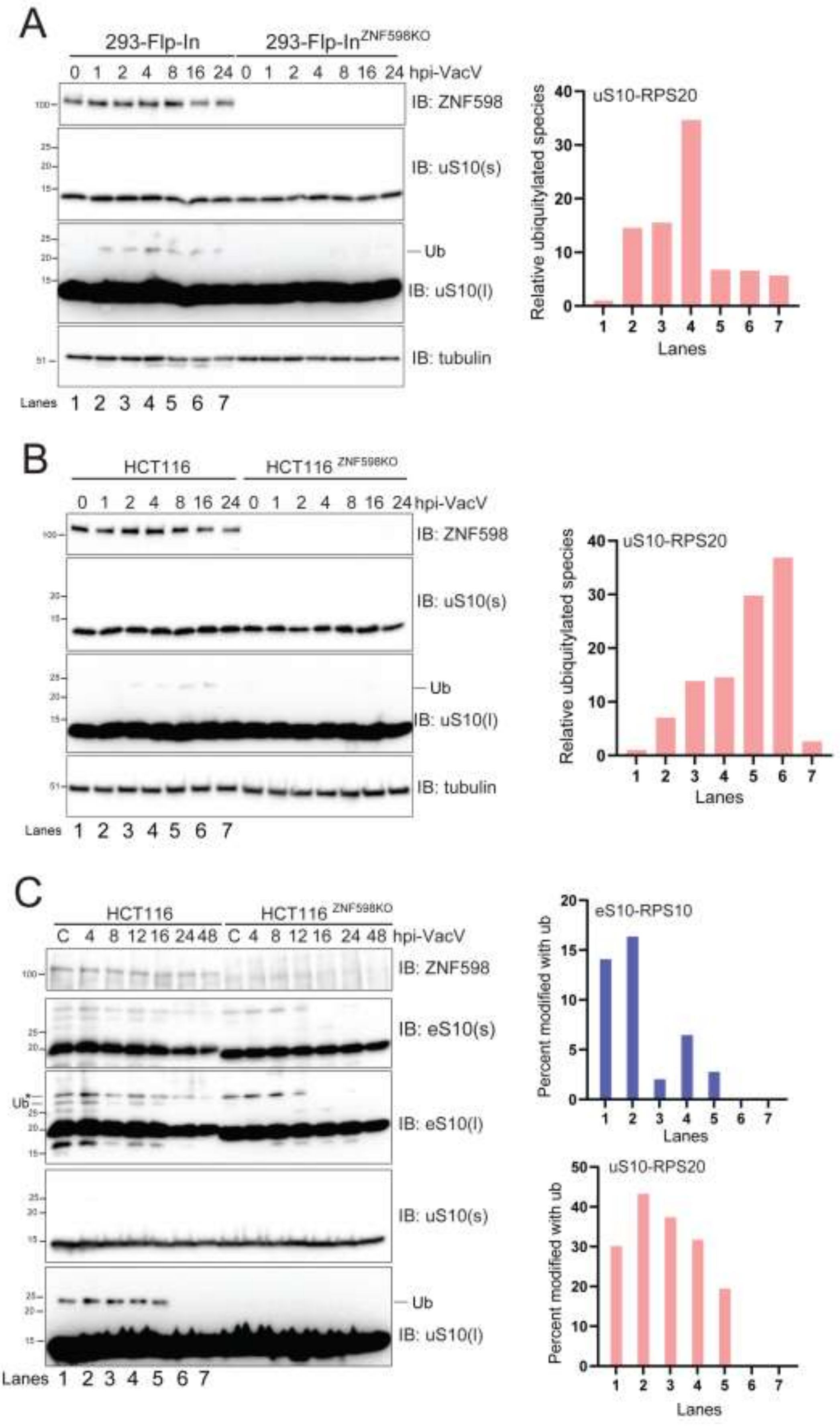

© 2021. Published by The Company of Biologists under the terms of the Creative Commons Attribution License (https://creativecommons.org/licenses/by/4.0/).

Second decision letter

MS ID#: JOCES/2020/257188

MS TITLE: Ribosome quality control activity potentiates vaccinia virus protein synthesis during infection

AUTHORS: Elayanambi Sundaramoorthy, Andrew P Ryan, Amit Fulzele, Marilyn Leonard, Matthew Daugherty, and Eric J Bennett
ARTICLE TYPE: Research Article

We have now reached a decision on the above manuscript.

To see the reviewers' reports and a copy of this decision letter, please go to: https://submit-jcs.biologists.org and click on the 'Manuscripts with Decisions' queue in the Author Area. (Corresponding author only has access to reviews.)

As you will see, the reviewers gave favourable reports but reviewer 2 raises some important points concerning interpretation of your data in light of previous literature that will require amendments to your manuscript. I hope that you will be able to carry these out, because I would like to be able to accept your paper.

We are aware that you may be experiencing disruption to the normal running of your lab that makes experimental revisions challenging. If it would be helpful, we encourage you to contact us to discuss your revision in greater detail. Please send us a point-by-point response indicating where you are able to address concerns raised (either experimentally or by changes to the text) and where you will not be able to do so within the normal timeframe of a revision. We will then provide further guidance. Please also note that we are happy to extend revision timeframes as necessary.

Please ensure that you clearly highlight all changes made in the revised manuscript. Please avoid using 'Tracked changes' in Word files as these are lost in PDF conversion.

I should be grateful if you would also provide a point-by-point response detailing how you have dealt with the points raised by the reviewers in the 'Response to Reviewers' box. Please attend to all of the reviewers' comments. If you do not agree with any of their criticisms or suggestions please explain clearly why this is so.

Reviewer 1

Advance summary and potential significance to field

See the comment of the first version

Comments for the author

I am good with he current revision.

Reviewer 2

Advance summary and potential significance to field

This manuscript will be an important contribution to the field but there are several critical issues that need to be resolved prior to publication. In light of the new data added the proposed model needs to be clarified, as it is now confusing. Many of the interpretations of apparent discrepancies with prior literature are simply misunderstandings on the part of the authors, for reasons explained in detail below; I hope this helps the authors, or perhaps they can provide a better rationalization

© 2021. Published by The Company of Biologists under the terms of the Creative Commons Attribution License (https://creativecommons.org/licenses/by/4.0/).

of their own interpretations. In fact, in my view prior studies largely align with and support the authors conclusions but presenting them in this inaccurate and unfair manner will confuse readers and muddy the field, unnecessarily.

Comments for the author

Main issues:
New data for uS10-Ub in figure 2C and S1, also shown and quantified as a reviewer figure in the rebuttal creates some confusion that needs to be clarified. First, the effects seem quite erratic rather than reproducible as stated, and the quantification shown to reviewers should be added to the main manuscript (with error bars for any repeats that might have been doen) to give a clearer sense of how variable the response is to readers – variability in such a low abundance response is understandable, but it should clearly indicated.

Equally importantly, this data now suggests that uS10-Ub is strongly activated within 1h of infection, when there is certainly very little burden from viral protein production. This also peaks much earlier than the viral protein synthesis burden that really only begins to be detectable from 12h onwards and maxes out at 24-48 (as shown in figure 3, for example). Yet the uS10-Ub levels actually decline dramatically in this later timeframe of 24-48h. The mass spec data is in line with the known kinetics of VacV protein production, which really doesn't take hold for a few hours at least, and PKR activation and inhibition don't occur until these later times either (and is not studied here to make any conjectures about). The authors reporter assays also show that RQC activity is not affected until later stages. This new uS10-Ub data now makes the proposed model quite confusing and seemingly at odds with itself, and this should be clarified in the text.

This kinetic element to the uS10-Ub profile is also important as it may explain one of the many "apparent" discrepancies with published work; DiGiuseppe only looked at uS10-Ub at late times of infection (20h and at higher MOI, so kinetics go faster), which is logically at the time of peak protein production and virus replication. Given the loss of uS10-Ub that is seen here in all three examples shown for 24-48h timepoints this actually matches up with the prior study and there is no discrepancy. Please clarify this point as it strengthens the authors own findings, and avoids both misrepresenting prior studies and creating confusion in the field.

Presentation in Figure 4A-B of genes broken down into kinetic classes is informative, and I think there's an opportunity to make some important points here. Particularly as the other reviewer requested a more thorough discussion of this aspect to benefit readers, as it also relates to the apparent discrepancies with prior studies. I'd suggest considering some of the points below to help frame things in a more helpful and accurate way.

Mass spec data, which becomes more obvious in the newly formatted Figure 4A-B, seems to suggest a kinetic delay in infection as fewer early proteins are detected in ZNF598 KO cells at 12-16hpi that begins to catch up and normalize by 24-48hpi. Meanwhile, as early proteins normalize, the late proteins show a notable difference at 24-48hpi; they have a reasonably similar number of data points which makes it a good comparative data set. This again may explain the "apparent" discrepancies with the DiGiuseppe paper, which I don't think are true discrepancies either. That paper just looked at a select few proteins at a late timepoint; two late proteins and one early-intermediate (I3). The I3 protein showed far less of a difference than the late proteins, and led to a suggestion that polyA leaders might be involved; obviously that was just an hypothesis based on a limited number of proteins examined, and this new data suggests a revision to this thinking is needed. But purely on a data level, the mass spec data here is actually in line with those observations, but reveals the limitations of the prior study that just looked at a few proteins at a single late timepoint. Explaining this clearly would not only prevent readers from thinking there are gross inconsistencies in the field, but it would add significance to this study that more thoroughly examined a wider range of proteins and timepoints and suggests there might be an overall kinetic delay that was not picked up by the other study.

This other study also detected no change in mRNA levels, albeit by conventional PCR approaches, but this also aligns with and supports the authors suggestion here that there are no major changes in viral gene transcription. While this is questioned by the other reviewer it is possible, given the sheer abundance of poxvirus proteins during infection, that smaller changes in abundance are not

© 2021. Published by The Company of Biologists under the terms of the Creative Commons Attribution License (https://creativecommons.org/licenses/by/4.0/).

sufficient to affect gene transcription to detectable extents but enough to reduce production of infectious virus.

The prior studies also suggested that there may be specificity to VacV 5' polyA leaders because the effects were specific to VacV, and not seen with other DNA or RNA viruses tested. While the study here suggests that idea does not withstand further testing, the authors also don't mention that other viruses have been tested when they suggest in the discussion that RQC may play a role in infection by other viruses. Again, the prior study is pretty limited in how many viruses were studied, but HSV-1 for example is quite similar to poxvirus in terms of PKR regulation and protein burden. Some of this could also be mentioned in the text, and it doesn't rule out that other viruses might also benefit from RQC.

Statements about the role of interferon responses in prior studies also seem misunderstood and misrepresented as discrepancies, unless the authors can rationalize their points more clearly. Prior studies showed that ZNF598 represses ISG expression through FATylation of RIG-I in cells that retain active innate pathways, and not just by transient siRNA approaches as implied by the authors [please cite the Wang, Cell Rep, 2019 paper that was suggested in prior review but is still omitted, as it uses other approaches to show this function of ZNF598]. But transformed cell lines often have defects in innate pathways (e.g. cGAS), even though they can still respond to exogenous interferon through other pathways. Addition of IFN as the authors do here can activate ISG expression by many mechanisms beyond RIG-I, so the fact that IFN addition induces ISG expression in ZNF598 knockout cells is not contrary to these prior findings and is not unexpected. It's actually still not clear what the point of this data actually is, as the authors don't add IFN or measure ISGs in the context of infection. So the relevance of these IFN responses to RQC during infection, the point of this study, is unclear. All the data shows is that the cells can respond to IFN without ZNF598 being present.

By contrast, the prior studies that are framed incorrectly as conflicting are in fact not only not conflicting they did the more direct experiment in the context of infection and clearly explained that they used HCT116 cells precisely because they lack a ZNF598-based interferon response to infection; they measure ISGs in HCT116 cells during infection and show no response. This was actually a key point of this prior paper, to show antiviral responses do not explain defects in VacV infection caused by loss of ZNF598 in HCT116 cells. This is completely disregarded by the authors. This should be acknowledged properly, not as a discrepancy but as actual support for the authors same conclusion.

I am not sure what the relevance of the luciferase assays are in Figure 4F as they seem to be done in uninfected cells, according to the legends and experimental description. Infection activates and alters polyA leader usage (as shown in 4E), and infection is dependent on RQC factors, not their loss. What does no effect of loss of RQC factors on polyA tell us without infection of the cells? The data is fine to keep in there but the text should be modified to clarify what this data actually means, and clarifying that it doesn't relate to translational control during infection.

Minor points are listed below but overall, I think the findings here are interesting and important additions to the field, but I think the authors fail to discuss prior studies in a fair or balanced manner, or in sufficient detail for readers unless they feel otherwise?

Other more minor issues:
A few points that would be helpful to clarify about the mass spec data and presentation for a general audience. In figure 3B-C, how do you calculate a H:L ratio between infected and uninfected cells for viral proteins that are not present in the uninfected cells? Is this a total protein ratio of some kind? I understand the reason for using Log2 scale but the actual changes in protein abundance are not so obvious to the eye on these graphs because of the scale and how close the mean lines are on these scales. Perhaps the actual difference could be placed above the graphs in figure 3-4 for ease of understanding; are we talking 30%? In figure 4C, how can there be a comparison of so many viral early and late proteins at 12h when in figure 4A-B there are far fewer viral proteins at this timepoint in ZNF598 KO cells? Is this a different data set?

In Figure 1B, the enhanced induction of uS10-Ub in S51A mutant MEFs by UV treatment remains pretty unconvincing. It would be helpful to add the quantification that is provided as a "reviewer figure". For that quantification, by eye the bar heights don't seem to align well with what is being

© 2021. Published by The Company of Biologists under the terms of the Creative Commons Attribution License (https://creativecommons.org/licenses/by/4.0/).

quantified e.g. differences in uS10-Ub in lanes 1-4 seem very different to levels shown for the quantification. Error bars for replicate measurements are needed. Quantification could also be added for Figure 1C given that again, effects are pretty small.

If I am not mistaken, the statement “RQC pathway components have not been demonstrated to play a role during translation initiation” may offend the Sonenberg Lab, who showed that ZNF598 regulates 4E-HP/GIGY and eIF4E/cap-dependent initiation, based on their 2012 paper?

Second revision

Author response to reviewers' comments

Response to Reviewer #2

This manuscript will be an important contribution to the field but there are several critical issues that need to be resolved prior to publication. In light of the new data added the proposed model needs to be clarified, as it is now confusing. Many of the interpretations of apparent discrepancies with prior literature are simply misunderstandings on the part of the authors, for reasons explained in detail below; I hope this helps the authors, or perhaps they can provide a better rationalization of their own interpretations. In fact, in my view prior studies largely align with and support the authors conclusions but presenting them in this inaccurate and unfair manner will confuse readers and muddy the field, unnecessarily.

We agree with reviewer 2 that our study is largely consistent with the one previous study (DiGiuseppe et al) that is main subject of this reviewer’s concerns. We feel that we have appropriately highlighted where our studies agree, which is the main point that ZNF598 and RQC (namely ribosomal ubiquitylation) is needed for optimal VacV replication. However, the previous study did invoke a mechanism (i.e. that polyA sequences within 5’UTRs of late VacV transcripts represent a challenge for the RQC pathway) to explain this dependence that was not investigated beyond an association with three vaccinia genes. Our purpose was to test this idea put forth in the previous study. We agree that proposing possible mechanisms is not a statement of fact. However, the entire scientific endeavor is rooted on experimental validation of these hypotheses and we see our study as expanding on the observations of that previous work to more clearly define the role that RQC plays during virus replication.

We apologize if the presentation of our data appeared unfair to this previous study. We have edited our manuscript (see highlighted sections) to attempt to remove and alter statements that the reviewer may have found inaccurate or unfair to avoid confusion.

Reviewer 2 Comments for the Author:

Main issues:

New data for uS10-Ub in figure 2C and S1, also shown and quantified as a reviewer figure in the rebuttal creates some confusion that needs to be clarified. First, the effects seem quite erratic rather than reproducible as stated, and the quantification shown to reviewers should be added to the main manuscript (with error bars for any repeats that might have been doen) to give a clearer sense of how variable the response is to readers – variability in such a low abundance response is understandable, but it should clearly indicated.

We generally try and avoid overt quantification of immunoblotting as these methods can be highly variable. We provided these figures to reviewers to help with interpretation.
While we can include them in the final version, our preference would be to omit them. We have provided infection time courses across three experiments in two different ZNF598 KO cell lines to provide readers a sense of both the variability and the extent of the ribosomal

© 2021. Published by The Company of Biologists under the terms of the Creative Commons Attribution License (https://creativecommons.org/licenses/by/4.0/).

modification.

Equally importantly, this data now suggests that uS10-Ub is strongly activated within 1h of infection, when there is certainly very little burden from viral protein production. This also peaks much earlier than the viral protein synthesis burden that really only begins to be detectable from 12h onwards and maxes out at 24-48 (as shown in figure 3, for example). Yet the uS10-Ub levels actually decline dramatically in this later timeframe of 24-48h. The mass spec data is in line with the known kinetics of VacV protein production, which really doesn't take hold for a few hours at least, and PKR activation and inhibition don't occur until these later times either (and is not studied here to make any conjectures about). The authors reporter assays also show that RQC activity is not affected until later stages. This new uS10-Ub data now makes the proposed model quite confusing and seemingly at odds with itself, and this should be clarified in the text.

Indeed, uS10 ubiquitylation can be detected as early as 1 hour post infection AND continues to be seen up to 16 hours post infection (and 24 hours in figure 2C). As we are unable to detect changes in protein expression until later time points to allow VacV protein production to reach detectable levels, we cannot say what impact this early RQC activation is having on VacV protein production before 8 hours post infection. Where we disagree, is that this data is at odds with what is described in the paper. We propose that VacV infection puts pressure on the RQC pathway, evident by its activation, and that when the pathway is inactivated, viral protein production in impaired. The fact that RQC activation happens early and sustains to time points where we see clear reduction in viral protein production in ZNF598-KO or uS10-KI cells (8 hours onward, see figure 2C,F) is in line with how it is described in the text. Further, ribosome-sequencing (ribosome profiling) data from VacV infected cells demonstrated that the number of ribosome- protected fragments from viral mRNAs was in excess of 40% of all reads (host+virus) at 2 hours post infection and reached 80% after 8 hours (PMID: 25903347). These observations argue that translation of substantial amounts of viral mRNA may occur at early time points, which we argue elict an RQC response.

This kinetic element to the uS10-Ub profile is also important as it may explain one of the many "apparent" discrepancies with published work; DiGiuseppe only looked at uS10-Ub at late times of infection (20h and at higher MOI, so kinetics go faster), which is logically at the time of peak protein production and virus replication. Given the loss of uS10-Ub that is seen here in all three examples shown for 24-48h timepoints, this actually matches up with the prior study and there is no discrepancy. Please clarify this point as it strengthens the authors own findings, and avoids both misrepresenting prior studies and creating confusion in the field.

We are unclear as to what discrepancy the reviewer is referring to. The previous study did not see any induction in ribosomal ubiquitylation and we did not reference this observation. It is entirely possible that the previous study used time points that would not allow them to visualize ribosome ubiquitylation. Indeed, one advantage of our study is we examined proteome and transcriptome dynamics at earlier time points to try and detect when VacV protein production is impacted by RQC loss.

Presentation in Figure 4A-B of genes broken down into kinetic classes is informative, and I think there's an opportunity to make some important points here. Particularly as the other reviewer requested a more thorough discussion of this aspect to benefit readers, as it also relates to the apparent discrepancies with prior studies. I'd suggest considering some of the points below to help frame things in a more helpful and accurate way.

Mass spec data, which becomes more obvious in the newly formatted Figure 4A-B, seems to suggest a kinetic delay in infection as fewer early proteins are detected in ZNF598 KO cells at 12-16hpi that begins to catch up and normalize by 24-48hpi. Meanwhile, as early proteins normalize, the late proteins show a notable difference at 24-48hpi; they have a reasonably similar number of data points which makes it a good comparative data set. This again may explain the "apparent" discrepancies with the DiGiuseppe paper, which I don't think are true discrepancies either. That paper just looked at a select few proteins at a late timepoint; two late proteins and one early-intermediate (I3). The I3 protein showed far less of a difference than the late proteins, and led to a suggestion that polyA leaders might be involved; obviously that was just an hypothesis based on a limited number of proteins examined, and this new data suggests a revision to this thinking is

© 2021. Published by The Company of Biologists under the terms of the Creative Commons Attribution License (https://creativecommons.org/licenses/by/4.0/).

needed. But purely on a data level, the mass spec data here is actually in line with those observations, but reveals the limitations of the prior study that just looked at a few proteins at a single late timepoint. Explaining this clearly would not only prevent readers from thinking there are gross inconsistencies in the field, but it would add significance to this study that more thoroughly examined a wider range of proteins and timepoints, and suggests there might be an overall kinetic delay that was not picked up by the other study.

Again, with apologies, I am unclear as to what the underlying issue is. The fact that the previous study only looked at three VacV proteins, prompted us to take a broader approach. I am uncertain why the reviewer thinks we have highlighted “gross inconsistences,” as we are merely pointing out what was proposed in the previous study and testing that proposed mechanism. The idea of a kinetic delay is entirely supported by the data. We have reinforced this idea in the discussion by saying, “Our data suggest there is an early kinetic delay in viral protein production in RQC deficient cells that likely first impacts early protein synthesis and is further exacerbated when late protein production becomes evident.”

The prior studies also suggested that there may be specificity to VacV 5’ polyA leaders because the effects were specific to VacV, and not seen with other DNA or RNA viruses tested. While the study here suggests that idea does not withstand further testing, the authors also don’t mention that other viruses have been tested when they suggest in the discussion that RQC may play a role in infection by other viruses. Again, the prior study is pretty limited in how many viruses were studied, but HSV-1 for example is quite similar to poxvirus in terms of PKR regulation and protein burden. Some of this could also be mentioned in the text, and it doesn’t rule out that other viruses might also benefit from RQC.

We have added this statement to the discussion, “While RQC deficiency did not impact HSV-1 or VSV replication (DiGiuseppe et al., 2018), it is possible other viruses that alter ISR responses are conditionally dependent on the RQC pathway for propagation.”

Statements about the role of interferon responses in prior studies also seem misunderstood and misrepresented as discrepancies, unless the authors can rationalize their points more clearly. Prior studies showed that ZNF598 represses ISG expression through FATylation of RIG-I in cells that retain active innate pathways, and not just by transient siRNA approaches as implied by the authors [please cite the Wang, Cell Rep, 2019 paper that was suggested in prior review but is still omitted, as it uses other approaches to show this function of ZNF598]. But transformed cell lines often have defects in innate pathways (e.g. cGAS), even though they can still respond to exogenous interferon through other pathways. Addition of IFN as the authors do here can activate ISG expression by many mechanisms beyond RIG-I, so the fact that IFN addition induces ISG expression in ZNF598 knockout cells is not contrary to these prior findings and is not unexpected. It’s actually still not clear what the point of this data actually is, as the authors don’t add IFN or measure ISGs in the context of infection. So the relevance of these IFN responses to RQC during infection, the point of this study, is unclear. All the data shows is that the cells can respond to IFN without ZNF598 being present.

Because loss of ZNF598 has been demonstrated to activate interferon signaling pathways and ISG expression, it was critical that we establish if ISG expression was not broadly induced in RQC deficient cell lines. Also, the ZNF598-dependent effects on ISG expression have been shown to NOT require its ligase activity, leaving it uncertain if ZNF598’s role during RQC (which is entirely dependent on its ligase activity and ribosomal ubiquitylation) has any impact on ISG signaling. Further, as was stated in the DiGiuseppe study, “we noted that many transformed cell lines are defective in interferon signaling,” we wanted to test if IFN addition could stimulate ISG expression. We agree that this does not address whether ISG expression is impacted upon VacV infection. Indeed, our RNAseq data shows that parental cells do not broadly induce ISG expression upon VacV infection. Further, our RNAseq dataset reveals the few ISGs that are induced upon VacV infection in parental cells (UBA7, PLEKHA4, and REC8) are also induced in uS10-KI cells. We feel these experiments are not only justified by the previous data, but omission of these experiments would leave it uncertain if the observed VacV replication reduction in RQC deficient cells was due to possible confounding effects on ISG expression.

We have added the following to the text to both cite the requested study and to clarify our

© 2021. Published by The Company of Biologists under the terms of the Creative Commons Attribution License (https://creativecommons.org/licenses/by/4.0/).

rationale.

"Further, loss of ZNF598 expression enhances RIG-I-mediated signaling and the protein abundance several ISGs (Wang et al., 2019). However, all these ZNF598-dependent effects on ISG expression do not depend on its ligase activity and it is unclear if limiting RQC activity itself alters ISG expression. Based on these previous studies, we monitored ISG stimulation upon IFN-□ addition to examine if interferon signaling was constitutively activated in our RQC mutant cell lines".

By contrast, the prior studies that are framed incorrectly as conflicting are in fact not only not conflicting, they did the more direct experiment in the context of infection and clearly explained that they used HCT116 cells precisely because they lack a ZNF598-based interferon response to infection; they measure ISGs in HCT116 cells during infection and show no response. This was actually a key point of this prior paper, to show antiviral responses do not explain defects in VacV infection caused by loss of ZNF598 in HCT116 cells. This is completely disregarded by the authors. This should be acknowledged properly, not as a discrepancy but as actual support for the authors same conclusion.

This is now acknowledged and cited in our manuscript. We also measured VacV alterations in gene expression as noted by our point above. We are again, unclear why the reviewer thinks we are being unfair to a single previous study. We are merely pointing out the previous findings and independently testing if altered ISG signaling contributes to the observed effects.

I am not sure what the relevance of the luciferase assays are in Figure 4F as they seem to be done in uninfected cells, according to the legends and experimental description. Infection activates and alters polyA leader usage (as shown in 4E), and infection is dependent on RQC factors, not their loss. What does no effect of loss of RQC factors on polyA tell us without infection of the cells? The data is fine to keep in there but the text should be modified to clarify what this data actually means, and clarifying that it doesn't relate to translational control during infection.

We have noted in the text that these experiments were done in the absence of infection. The relevance of these assays is to directly test if polyA sequences in the 5'UTR impact translation in an RQC-dependent manner. As polyA sequences DO disrupt translation of downstream genes in an RQC-dependent manner when present within the coding sequence, it was an open question whether RQC loss would also augment translation from genes containing polyA sequences within the 5'UTR. These studies are very relevant to VacV late gene expression.

Other more minor issues:A few points that would be helpful to clarify about the mass spec data and presentation for a general audience. In figure 3B-C, how do you calculate a H:L ratio between infected and uninfected cells for viral proteins that are not present in the uninfected cells? Is this a total protein ratio of some kind? I understand the reason for using Log2 scale but the actual changes in protein abundance are not so obvious to the eye on these graphs because of the scale and how close the mean lines are on these scales. Perhaps the actual difference could be placed above the graphs in figure 3-4 for ease of understanding; are we talking 30%? In figure 4C, how can there be a comparison of so many viral early and late proteins at 12h when in figure 4A-B there are far fewer viral proteins at this timepoint in ZNF598 KO cells? Is this a different data set?

A background ion intensity in a window that brackets the measured ion pair is calculated and used as the denominator when one of the signals (light in this case) is absent. This is common practice in quantitative MS experiments. Otherwise very large changes (like expression of viral proteins) would be omitted from all proteomic studies. For consistency, we do not feel there is a need to replot all MS data. The data that is represented in figures 3 and 4 is unchanged. However, in figure 4C, the difference in the log2 ratios is depicted so the number of points is the sum of the proteins detected in both ZNF598 KO and parental cells. Issues arising from this type of comparing ratio of ratios is why we changed the labeling scheme for the experiments using the uS10-KI cells to allow direct comparison of VacV proteins between the genotypes.

In Figure 1B, the enhanced induction of uS10-Ub in S51A mutant MEFs by UV treatment remains pretty unconvincing. It would be helpful to add the quantification that is provided as a "reviewer figure". For that quantification, by eye the bar heights don't seem to align well with what is being

© 2021. Published by The Company of Biologists under the terms of the Creative Commons Attribution License (https://creativecommons.org/licenses/by/4.0/).

quantified e.g. differences in uS10-Ub in lanes 1-4 seem very different to levels shown for the quantification. Error bars for replicate measurements are needed. Quantification could also be added for Figure 1C given that again, effects are pretty small.

We generally try and avoid overt quantification of immunoblotting as these methods can be highly variable. We provided these figures to reviewers to help with interpretation.
While we can include them in the final version, our preference would be to omit them. Indeed, the eIF2a phosphorylation response is variable. We sought to both capture this variability and established robustness by using two different ISR agonists and two different ways to perturb eIF2a signaling (mutant MEFs and ISRIB).

If I am not mistaken, the statement “RQC pathway components have not been demonstrated to play a role during translation initiation” may offend the Sonenberg Lab, who showed that ZNF598 regulates 4E-HP/GIGY and eIF4E/cap-dependent initiation, based on their 2012 paper?

I am uncertain what to take from this statement. We have rephrased this statement to clarify the point we were trying to make. Previous work by the Sonenberg lab has shown that ZNF598 regulation of 4EHP/GIGYF1/2 is independent of its ligase activity. Indeed, ZNF598 directly binds to 4EHP and GIGYF1/2 as nicely demonstrated by the referenced study and detailed by Vind et al (PMID: 32289254) who again show that this impact was not ligase dependent. To date, there is no established role for ribosomal ubiquitylation directly impacting scanning or initiation outside of effects on eIF2a signaling. We now clarify this issue to specifically call attention to ligase-dependent ZNF598 activities by stating: “While recent reports have demonstrated that elongation collisions can result in feedback inhibition of translation initiation by various mechanisms (Hickey et al., 2020; Juszkiewicz et al., 2020a; Meydan and Guydosh, 2020b; Sinha et al., 2020; Vind et al., 2020b; Wu et al., 2020; Yan and Zaher, 2021) these pathways act largely in manner that does not require ZNF598 activity nor ribosome ubiquitylation. Consistent with the lack of an established direct role for ZNF598 or uS10 or eS10 ubiquitylation during ribosome scanning prior to elongation, our data indicates that mRNAs containing non-coding polyA sequences within the 5’UTR, while broadly repressive, are not translated more efficiently in cells with defective RQC activity.

We believe it is important to make this point as it addresses another model that was put forth in the DiGiuseppe paper. In that paper, they state “Perhaps most surprising is the stimulatory role that ZNF598 plays in poxvirus translation, which also suggests a role for ZNF598 during scanning. Although limited studies of ZNF598 to date have focused on its role in 80S stalling, its primary substrates are RPSs and a large fraction of the cellular pool of ZNF598 co-sediments with initiation factors and 40S subunits (Garzia et al., 2017, Sundaramoorthy et al., 2017). As such, roles in scanning are very plausible, at least in contexts where poly(A) is present in 5′ UTRs.” Given the ligase dependence, which has previously been shown to not play any role in initiation, and the lack of dependence on polyA sequences within the 5’UTR, we wished to clarify a model that is consistent with all of the available literature.

Third decision letter

MS ID#: JOCES/2020/257188

MS TITLE: Ribosome quality control activity potentiates vaccinia virus protein synthesis during infection

AUTHORS: Elayanambi Sundaramoorthy, Andrew P Ryan, Amit Fulzele, Marilyn Leonard, Matthew Daugherty, and Eric J Bennett
ARTICLE TYPE: Research Article

I am happy to tell you that your manuscript has been accepted for publication in Journal of Cell Science, pending standard ethics checks.

© 2021. Published by The Company of Biologists under the terms of the Creative Commons Attribution License (https://creativecommons.org/licenses/by/4.0/).
